# The genetic interacting landscape of 63 candidate genes in Major Depressive Disorder: an explorative study

**DOI:** 10.1186/1756-0381-7-19

**Published:** 2014-09-09

**Authors:** Magnus Lekman, Ola Hössjer, Peter Andrews, Henrik Källberg, Daniel Uvehag, Dennis Charney, Husseini Manji, John A Rush, Francis J McMahon, Jason H Moore, Ingrid Kockum

**Affiliations:** 1 Department of Clinical Neuroscience, Neuroimmunology Unit, Karolinska Institutet, Stockholm, Sweden; 2 Department of Mathematics, Stockholm University, Stockholm, Sweden; 3 Institute of Quantitative Biomedical Science, Department of Genetics and Community and Family Medicine, Geisel School of Medicine, Dartmouth College, Hanover, NH, USA; 4 Institute of Environmental Medicine, Karolinska Institutet, Stockholm, Sweden; 5 Department of Psychiatry, Mount Sinai School of Medicine, New York, NY, USA; 6 Laboratory of Molecular Pathophysiology, NIMH, NIH, Department of Health & Human Services (DHHS), Bethesda, USA; 7 Academic Medical Research Institute, Duke-National University of Singapore, Singapore, Singapore; 8 Genetic Basis of Mood & Anxiety Disorders Section, Human Genetic Branch, NIMH, NIH, Department of Health & Human Services (DHHS), Bethesda, USA

**Keywords:** Additive interaction, Multiplicative interaction, Logistic regression, Data mining and machine learning, Major depressive disorder

## Abstract

**Background:**

Genetic contributions to major depressive disorder (MDD) are thought to result from multiple genes interacting with each other. Different procedures have been proposed to detect such interactions. Which approach is best for explaining the risk of developing disease is unclear.

This study sought to elucidate the genetic interaction landscape in candidate genes for MDD by conducting a SNP-SNP interaction analysis using an exhaustive search through 3,704 SNP-markers in 1,732 cases and 1,783 controls provided from the GAIN MDD study. We used three different methods to detect interactions, two logistic regressions models (multiplicative and additive) and one data mining and machine learning (MDR) approach.

**Results:**

Although none of the interaction survived correction for multiple comparisons, the results provide important information for future genetic interaction studies in complex disorders. Among the 0.5% most significant observations, none had been reported previously for risk to MDD. Within this group of interactions, less than 0.03% would have been detectable based on main effect approach or an a priori algorithm. We evaluated correlations among the three different models and conclude that all three algorithms detected the same interactions to a low degree. Although the top interactions had a surprisingly large effect size for MDD (e.g. additive dominant model P_uncorrected_ = 9.10E-9 with attributable proportion (AP) value = 0.58 and multiplicative recessive model with P_uncorrected_ = 6.95E-5 with odds ratio (OR estimated from β3) value = 4.99) the area under the curve (AUC) estimates were low (< 0.54). Moreover, the population attributable fraction (PAF) estimates were also low (< 0.15).

**Conclusions:**

We conclude that the top interactions on their own did not explain much of the genetic variance of MDD. The different statistical interaction methods we used in the present study did not identify the same pairs of interacting markers. Genetic interaction studies may uncover previously unsuspected effects that could provide novel insights into MDD risk, but much larger sample sizes are needed before this strategy can be powerfully applied.

## Introduction

Major depressive disorder (MDD) is a common [1] complex [2] psychiatric disorder with major health concerns worldwide [3,4]. Heritability estimates of ~37% [5] implicate a partially genetic causation. This has motivated numerous linkage studies [6], association studies of candidate genes [7] and recently even a few genome-wide association studies (GWAS) [8]. However, convincing replication across independent studies is lacking, and no major MDD risk locus has been identified leaving much of the estimated heritability unexplained. This implies that MDD is genetically far more complex than previously expected, with influences from both allelic and locus heterogeneities in parallel with exposures to adverse social events and behavior, hampering success in identifying genetic risk factors [6,7,9] within this heterogeneous syndrome.

A recent mega-analysis shows that it is unlikely that MDD is caused by a few genetic risk factors with large effect [8]. The explained genetic variance is still low which opens up for the possibility that interaction effects from both genetic and environmental risk factors are important source for explaining the remaining heritability [10-14]. The role of interactions (also known as epistasis or synergy) as a versatile mechanism for regulating health and disease has been proposed [15-18], and has recently been suggested to increase risk for schizophrenia [19,20] and bipolar disorder [21]. Increasing the sample size alone and using a single gene model will not reveal the complete genetic architecture of MDD. Detailed characterization of the interacting landscape will thus be a critical factor in understanding why people are at risk for this debilitating disorder [22,23].

Numerous methods are available for detecting gene-gene interactions [24-26], each of which has been designed with different assumptions of how biological interactions can be detected with statistical methods.

Historically, two statistical principles have evolved: one represents genetic model-based methods and one model-free method [27,28]. The model-based approaches are designed to calculate interactions based on linear regression [29]. The model-free systems use different applications of data mining and machine learning approaches [30,31]. It is not yet clear which model is best at identifying interacting factors involved in disease susceptibility.

For this study we stated two hypotheses. Our first hypothesis was that common genetic risk factors in a set of candidate genes, act through a variety of interactions in the susceptibility for MDD. Our second hypothesis was that no single available statistical method would completely capture all possible forms of interactions. We therefore chose three different methods for modeling potential genetic interactions and examined to what extent these different statistical methods report synergy effects with relevance for susceptibility to MDD.

To address these questions we designed an incremental approach (analytic strategy in Additional file 1: Figure S1) with a reduction of genetic heterogeneity using two cohorts, to systematically examine SNP-interaction effects using both genetic model-based and model-free methods. The former consists of one additive and one multiplicative interaction method. The model-free method was a multifactor dimensionality reduction (MDR) approach.

The objective was not to compare the statistical methods for detecting interaction but we evaluated the methods in those situations where the combinations of pairs of tested SNPs were identical. Both study populations we used in this study have been used for genome-wide association analyses of MDD [32,33]. The candidate genes we used in this study have not been tested in a separate analysis previously.

## Methods and materials

### Study populations

The Sequenced Treatment Alternatives to Relive Depression (STAR*D) study [34] and the Genetic Association Information Network, GAIN major depressive disorder study [35] provided the two study populations. STAR*D subjects were outpatients from primary care or psychiatric clinics who met DSM-IV criteria for single or recurrent non-psychotic MDD, age 18–65 years, with no history of bipolar disorder, schizophrenia, schizoaffective disorder, or any other psychotic disorder. The STAR*D sample comprised of 634 controls matched with 1,256 cases. GAIN cases were recruited from four different longitudinal studies of patients with MDD in the Netherlands [32,36-38]. Inclusion criteria were life-time diagnosis of MDD (DSM-IV), and an age between 18–65 years. A sample of 1,822 biologically unrelated controls was matched with 1,821 cases. Full details of both study populations are available in Additional file 2: Supplementary Materials.

### Candidate gene selection SNP genotyping and quality control

The 68 candidate genes we used in this study were derived from in the STAR*D study and were prioritized on the basis of previous results of association to MDD, for association to response to antidepressant treatment [39] (Additional file 3: Table S1). Full details of genotyping, marker selection and QC analyses are provided in Additional file 2: Supplementary Materials.

We included markers that mapped within 10 kb of each candidate gene according to NCBI36/hg18. After QC analysis the STAR*D dataset consisted of 1,240 cases and 630 control and 654 SNP markers. The GAIN dataset consisted of 1,732 cases, 1,783 controls and 1,516 SNPs.

### Statistical power

Power calculations are conditional on estimates of the correct specifications of the underlying genetic model. Since such a ‘true’ model is not known, we allowed several model parameters to vary, including misspecification of the diagnosis. The mean power estimates for the single marker analyses were 53% in GAIN and 32% in STAR*D samples (Additional file 4: Table S2). Detailed power calculations are provided in Additional file 2: Supplementary Materials.

As a method for power calculation in the additive interaction method or the MDR approach is not validated we have not generated any estimates of power in the interaction analyses.

### Marker selection for SNP-SNP interaction analysis

For the logistic regression methods where it became unmanageable to test interactions between all markers, our approach was to prioritize markers according to predicted attributable dependencies [28] defined through main effect (P < 0.05, n = 43) in single marker analysis [40] and from markers with no-main effects (n = 49) using predicted dependencies based on a computational filtering approach implemented in the MDR software [41]. In total, for the two logistic regression methods, 92 markers were tested against the full dataset of 3,704 markers. For the genetic model-free MDR approach, we managed to include the full SNP-dataset using an exhaustive interaction method. We did not filter the markers for pair-wise linkage disequilibrium (LD) patterns. However, when presenting the top 10 interactions for each model LD pruning was performed with an r^2^ value > 0.2. Interaction analyses were only performed for autosomal markers because the current software versions do not handle chromosome X markers correctly.

### Statistical analyses

#### Single marker association analysis and meta-analysis

For autosomal markers association was tested using a Cochrane Armitage test (SNPtest) assuming an additive model, using the top 4 principal components as covariates. For chromosome X markers the Unphased software (additive allele-wise model for the log odds ratio) was used. Fixed effects (Mantel-Haenzel) or random effects (DerSimonian-Laird) meta-analyses were performed after Woolf’s test for heterogeneity with an applied threshold of P < 0.05, performed in R (rmeta-package). The single-marker analysis and meta-analysis was performed in order to assess a shared genetic background to MDD between the two study populations. We used nominal P value as our cutoff for defining homogeneity in association between the two study populations. The threshold for reporting association to MDD in the single marker analyses was estimated by a family-wise error rate (FWER), using Bonferroni’s method.

#### SNP-SNP interaction analysis

We considered three different algorithms for detecting SNP-SNP interactions. Interaction effects in the additive and multiplicative methods were tested using logistic regression implemented in a JAVA coded software designed to handle large datasets (coded in our own lab and available upon request) which is a modified version of the GEIRA software [42]. Testing for interaction effects using a machine learning and data mining approach was conducted in a MDR software [43]. See Additional file 2: Supplementary Materials for detailed descriptions of these algorithms. To examine the ability of the identified interactions to predict disease status, we calculated the sensitivity and specificity scores. The population attributable fraction (PAF) was calculated to quantify the proportion of MDD affected individuals attributed to the genetic risk (described in Additional file 2: Supplementary Materials).

Test for correlation between identified pair-wise set of markers between different statistical interaction methods was estimated by calculating Pearson’s r (carried out in R).

## Results

### Assessment of genetic heterogeneity between STAR*D and GAIN

We considered a nominal threshold of association (P < 0.05) for evaluating a shared genetic background in the 68 candidate genes to MDD between the two study populations.

Of the 173 SNPs that were genotyped in both STAR*D and GAIN none were found to be nominally associated in both study populations. Of the 34 SNPs found at P < 0.05 in STAR*D 10 markers were genotyped in GAIN.

Of the 57 SNPs with P < 0.05 in GAIN 3 markers were genotyped in STAR*D (Additional file 5: Figure S2A-B and Additional file 6: Table S3A-B). We imputed markers in GAIN for the 15 genes that contained nominally associated markers in STAR*D. This analysis generated 28 additional SNPs at P < 0.05 of which none overlapped with nominally associated markers in STAR*D (Additional file 6: Table S3A and C). None of the nominally associated markers in the two study populations were in high LD (r^2^ ≥ 0.8).

As a final step to test for heterogeneity we included both study populations and performed a meta-analysis. We selected the nominally associated SNPs in STAR*D that were identical with any marker in GAIN, genotyped or imputed (n = 15). However, only 10 markers had the same risk allele and only one marker was nominally associated in the meta-analysis, rs2137330 in the *NR3C2* gene, pooled OR = 1.13 and P = 0.039 (Additional file 5: Figure S2C).

A shared genetic risk between the two study-populations could not be confirmed. Therefore only the GAIN sample, which was the sample with more complete genotyping in the candidate genes, was used for the subsequent gene-gene interaction analyses.

### Overall interaction findings

Genotypes from 3,704 imputed or genotyped markers in the 63 tested candidate genes (chromosome X genes excluded and two autosomal genes without markers in GAIN) were tested for interaction effects. None of the tests generated P values that survived correction for multiple testing with permutation in either the logistic regression or MDR methods. Additional file 7: Figure S3A-C illustrates the complete set of interactions generated from the different methods that were used for the assessment of interaction effects. For each one of the linear models, 340,768 interactions were evaluated (testing 92 against 3,704 markers), whereas for the MDR method 3,704 markers were tested in an exhaustive interaction analysis.

The large number of tests performed penalized our approach with regard to report a significant interaction, and none of the tests generated a P value that survived correction for multiple testing.

In spite of the non-significant results there are interesting observations worth noting. Among the model-based methods, a large number of interactions were observed with P values of interaction below a nominal significance level (Additional file 7: Figure S3A-B). We therefore hypothesized, based on the notion that our study sample was markedly underpowered, that the 0.5% most significant interactions would be enriched for interactions involved in MDD-susceptibility. In a very conservative approach, we therefore next considered the top 10 ranked interactions (LD filtered, r^2^ ≤ 0.2) in each one of the different interaction methods as a source of information for better understanding of the genetic interacting landscape in these candidate genes for MDD-susceptibility.

Our next observation concerns the unrealistically large fraction of negative AP estimates. In some instances the observed AP values exceeded the theoretical limit of −1.0 (i.e. AP < −1.0). This observation prompted us to address the question if the negative AP values are valid estimates for the reduction of risk (Additional file 8: Figure S4A-D). We observed a skewing of AP values towards the negative side of the distribution and a correlation between large negative AP values and few individuals in one of the exposure groups. There did not appear to be a correlation between the lowest observed OR and negative AP, as AP < −1.0 was observed for both OR close to 1.0 and large OR’s. However there was a negative correlation between the largest observed single exposure OR and AP when AP < 0. We concluded that although these negative AP values may indicate negative interactions, their magnitudes are far too large to warrant statistical analysis. No deflecting pattern was observed among positive AP values.

### Top 10 interactions

Of the ten strongest interactions generated from the three different algorithms, none represents pairs of markers that have previous been reported to interact in the susceptibility to MDD. We also noted that within this group of interactions the markers did not represent those selected based on predictive interaction effects using main effect or an algorithm that predict involvements SNP-SNP interactions. Although the three different methods generated metrics of interaction effects with different assumptions and were thus not readily comparable, we further noted that the estimates of interaction effects were surprisingly strong for the regression models.

Among the ten most significant interactions in the dominant additive model, the effect measures of interaction (AP) were relatively strong (AP > 0.51, uncorrected P < 3.77E-5). The most significant interaction (uncorrected P = 9.10E-9, permuted P = 0.14) consisted of two markers located in the *ARGHAP10* gene, rs9332471 and rs6845865 (AP = 0.58) (Table 1A). The rs9332471 marker also shows interaction with another marker, rs2306910, in the *ARHGAP10* gene. These 3 markers are in a weak LD (r^2^ ≤ 0.2) with each other. This gene is involved in intracellular signaling and has a role in regulation of apoptosis. In the recessive model (Table 1B), the strongest signal was observed between markers located in the *GRIN2B* and *RNF20* genes (AP = 0.69, uncorrected P = 7.65E-7, permuted P = 0.56).

**Table 1:** Result of the 10 most nominally significant interactions

**A: Additive dominant**														
**Marker 1**			**Marker 2**			**Interaction statistics**								
											**Cases/Controls with marker 1 and marker 2 conferring risk (1) or no-risk (0)**			
								**Permuted**						
**Chr**	**Gene**	**Rs**	**Chr**	**Gene**	**Rs**	**AP (95% C.I.)**	**AP P**	**P**	**PAF**	**AUC**	**00**	**01**	**10**	**11**
4	ARHGAP10	rs9332471	4	ARHGAP10	rs6845865	0.58 (0.39:0.78)	9.10E-9	0.14	0.147	0.5153	1009/1073	169/174	475/500	78/33
6	HTR1E	rs6922679	16	GRIN2A	rs17570500	*0.71 (0.42:1.002)	1.92E-6	0.57	0.142	0.5046	48/50	20/6	1332/1388	310/321
4	ARHGAP10	rs9332471	4	ARHGAP10	rs2306910	0.63 (0.37:0.89)	2.40E-6	0.61	0.149	0.5136	1201/1273	190/182	233/238	35/12
4	ARHGAP10	rs9991394	4	ARHGAP10	rs12645249	0.59 (0.34:0.84)	2.58E-6	0.63	0.146	0.5173	1162/1257	332/326	154/158	47/18
9	GRIN3A	rs7873495	11	GRIK4	rs10892635	**-1.08 (-1.56:-0.59)	1.26E-5	0.90	0.139	0.5216	290/258	10/30	1308/1390	122/105
12	GRIN2B	rs12371702	9	GRIN3A	rs13292935	***-0.92 (-1.35:-0.50)	1.89E-5	0.95	0.146	0.5375	1003/1122	442/377	236/202	51/82
12	GRIN2B	rs11832404	4	NR3C2	rs982076	**-1.23 (-1.81:-0.65)	3.49E-5	0.99	0.142	0.5266	257/182	10/24	1268/1367	92/86
12	GRIN2B	rs11832404	11	GRIK4	rs7928347	**-1.27 (-1.89:-0.66)	5.24E-5	0.99	0.142	0.5300	255/185	7/18	1234/1332	87/78
4	ARHGAP10	rs9332471	9	SLC1A1	rs184204	0.53 (0.27:0.79)	6.28E-5	0.99	0.145	0.5157	1212/1292	194/183	271/282	53/23
5	GRIA1	rs17114975	12	GRIN2B	rs1012587	*0.64 (0.33:0.95)	6.54E-5	0.99	0.129	0.5084	83/79	24/8	1381/1448	241/245
**B: Additive recessive**														
**Marker 1**			**Marker 2**			**Interaction statistics**								
											**Cases/Controls with marker 1 and marker 2 conferring risk (1) or no-risk (0)**			
								**Permuted**						
**Chr**	**Gene**	**Rs**	**Chr**	**Gene**	**Rs**	**AP (95% C.I.)**	**AP P**	**P**	**PAF**	**AUC**	**00**	**01**	**10**	**11**
9	RNF20	rs16920473	12	GRIN2B	rs2216127	0.69 (0.42:0.97)	7.65E-7	0.56	0.149	0.5238	377/446	1296/1305	12/14	46/14
4	ARHGAP10	rs4835456	11	GRIK4	rs4936540	0.72 (0.42:1.02)	2.70E-6	0.72	0.148	0.5136	1105/1176	22/23	582/571	22/6
4	ARHGAP10	rs6824449	16	SLC6A2	rs192303	***-1.28 (-1.85:-0.72)	8.30E-6	0.86	0.149	0.5319	1293/1414	208/159	184/144	16/32
16	GRIN2A	rs4782040	11	GRIK4	rs11218032	***-0.99 (-1.43:-0.55)	8.73E-6	0.87	0.149	0.5211	1244/1306	122/126	250/207	9/32
12	GRIN2B	rs3764030	5	HTR4	rs6865654	0.68 (0.37:0.99)	1.27E-5	0.91	0.148	0.5063	1363/1423	47/48	231/235	22/7
11	GRIK4	rs7939968	4	NR3C2	rs12641471	0.52 (0.28:0.76)	2.03E-5	0.94	0.144	0.5170	1088/1165	192/186	275/292	69/32
4	ARHGAP10	rs6824449	16	GRIN2A	rs11640235	***-1.01 (-1.56:-0.55)	3.60E-5	0.98	0.149	0.5204	1355/1435	210/163	105/108	8/25
11	GRIK4	rs7939968	4	NR3C2	rs4835508	0.51 (0.27:0.75)	3.77E-5	0.98	0.143	0.5186	1029/1118	185/179	275/295	70/33
11	GRIK4	rs7939968	4	ARHGAP10	rs12641157	0.64 (0.33:0.94)	5.05E-5	0.99	0.148	0.5178	1347/1444	260/229	93/95	26/8
3	HTR3C	rs6766410	6	LAMA4	rs6913656	***-0.84 (-1.25:-0.43)	5.48E-5	0.99	0.148	0.5216	1210/1279	243/207	217/218	21/49
**C: Multiplicative dominant**														
**Marker 1**			**Marker 2**			**Interaction statistics**								
											**Cases/Controls with marker 1 and marker 2 conferring risk (1) or no-risk (0)**			
								**Permuted**						
**Chr**	**Gene**	**Rs**	**Chr**	**Gene**	**Rs**	**OR (95% C.I.)**	**P**	**P**	**PAF**	**AUC**	**00**	**01**	**10**	**11**
4	GRIA2	rs17244157	16	GRIN2A	rs1861192	0.44 (0.30:0.64)	2.39E-5	0.85	0.120	0.5442	712/838	175/120	589/539	98/120
9	SLC1A1	rs10974611	6	LAMA4	rs2032568	0.47 (0.38:0.67)	3.60E-5	0.92	0.125	0.5389	536/624	154/116	699/682	145/195
12	GRIN2B	rs12371702	9	GRIN3A	rs13292935	0.41 (0.26:0.62)	3.72E-5	0.95	0.114	0.5375	1003/1122	442/377	236/202	51/82
4	GRIA2	rs17244157	16	GRIN2A	rs16966731	0.45 (0.31:0.66)	3.78E-5	0.95	0.127	0.5322	572/642	147/88	798/808	135/162
21	GRIK1	rs379182	11	GRIK4	rs17124632	0.24 (0.12:0.48)	4.41E-5	0.97	0.148	0.5244	80/111	46/14	1165/1221	357/342
10	RPP30	rs11186343	7	HTR5A	rs2919435	2.49 (1.60:3.89)	5.51E-5	0.98	0.130	0.5295	145/97	58/89	951/1028	558/555
3	NR1I2	rs6438549	12	GRIN2B	rs12823982	0.45 (0.30:0.67)	7.17E-5	0.99	0.114	0.5333	927/1011	222/182	505/473	67/105
13	HTR2A	rs666693	22	COMT	rs8185002	3.05 (1.75:5.31)	8.82E-5	0.99	0.148	0.5325	596/548	34/61	927/1011	83/58
4	NR3C2	rs7691663	5	GRIA1	rs1422897	2.09 (1.44:3.02)	1.02E-4	0.99	0.110	0.5310	124/85	445/490	185/237	977/964
19	GRIK5	rs4803523	5	GRIA1	rs13359392	2.31 (1.51:3.55)	1.27E-4	1.0	0.144	0.5350	548/487	58/90	962/1068	161/135
**D: Multiplicative recessive**														
**Marker 1**			**Marker 2**			**Interaction statistics**								
											**Cases/Controls with marker 1 and marker 2 conferring risk (1) or no-risk (0)**			
								**Permuted**						
**Chr**	**Gene**	**Rs**	**Chr**	**Gene**	**Rs**	**OR (95% C.I.)**	**P**	**P**	**PAF**	**AUC**	**00**	**01**	**10**	**11**
5	GRIA1	rs574071	11	HTR3B	rs1672717	0.52 (0.39:0.71)	2.72E-5	0.70	0.132	0.5433	371/473	701/646	222/182	305/346
4	NR3C2	rs7691663	5	GRIA1	rs9686702	1.84 (1.38:2.44)	3.28E-5	0.76	0.117	0.5375	270/211	313/374	445/516	703/679
3	GSK3B	rs1719889	16	GRIN2A	rs1102967	0.55 (0.41:0.73)	3.30E-5	0.76	0.139	0.5423	228/345	569/517	308/289	534/552
11	GRIA4	rs17104807	22	COMT	rs2020917	2.39 (1.57:3.63)	4.56E-5	0.86	0.118	0.5386	98/86	643/719	86/149	848/784
12	GRIN2B	rs3764030	16	GRIN2A	rs1868289	4.99 (2.26:11.03)	6.95E-5	0.96	0.133	0.5169	41/13	29/44	695/742	948/970
5	GRIA1	rs574071	16	GRIN2A	rs1070548	2.21 (1.48:3.31)	1.17E-4	0.99	0.142	0.5284	517/532	819/860	70/110	186/135
9	NTRK2	rs1624327	16	GRIN2A	rs1097784	1.94 (1.38:2.72)	1.19E-4	0.99	0.138	0.5332	562/581	639/662	128/186	237/178
4	ARHGAP10	rs6824449	16	SLC6A2	rs192303	0.27 (0.14:0.54)	1.68E-4	1.0	0.119	0.5319	1293/1414	208/159	184/144	16/32
6	GRIK2	rs7770500	6	LAMA4	rs2072019	2.06 (1.41:3.00)	1.76E-4	1.0	0.116	0.5133	139/102	666/719	119/164	792/781
9	SLC1A1	rs7022369	5	GRIA1	rs12658202	1.92 (1.36:2.71)	1.98E-4	1.0	0.067	0.5285	100/147	281/326	399/352	957/952
**E: MDR**														
**Marker 1**			**Marker 2**			**Interaction statistics**								
										**Cases/Controls with genotype combination at predicted risk group**				
										
**Chr**	**Gene**	**Rs**	**Chr**	**Gene**	**Rs**	**Balanced accuracy**	**PAF**	**OR (95% C.I.)**	**P**	**High risk**	**Low risk**	**TPR**	**FPR**	
12	GRIN2B	rs1012587	3	HTR3C	rs6762938	0.5513	0.0980	1.51 (1.32:1.73)	1.168E-09	907/751	825/1032	0.524	0.421	
16	GRIN2A	rs8045893	3	HTR3C	rs6762938	0.5498	0.0900	1.49 (1.30:1.71)	4.452E-09	1033/887	699/896	0.596	0.497	
6	HTR1E	rs17222848	5	GRIA1	rs17515709	0.5480	0.0950	1.47 (1.28:1.68)	1.707E-08	933/790	799/993	0.539	0.443	
11	GRIA4	rs609665	6	GRIK2	rs2518171	0.5476	0.0917	1.46 (1.28:1.68)	2.051E-08	982/842	750/941	0.567	0.472	
6	GRIK2	rs17828670	3	GSK3B	rs6771023	0.5470	0.0894	1.46 (1.28:1.67)	2.757E-08	1021/884	711/899	0.589	0.554	
13	HTR2A	rs9526240	11	BDNF	rs6265	0.5468	0.0912	1.45 (1.27:1.66)	3.643E-08	984/847	748/936	0.568	0.475	
16	SLC6A2	rs1814270	6	FKBP5	rs9462104	0.5465	0.1020	1.46 (1.28:1.68)	3.031E-08	809/668	923/1115	0.467	0.375	
13	HTR2A	rs17288723	9	GRIN3A	rs11788456	0.5454	0.0937	1.44 (1.26:1.64)	8.039E-08	936/802	796/981	0.540	0.500	
12	GRIN2B	rs10845847	12	GRIN2B	rs12301788	0.5454	0.0809	1.48 (1.29:1.71)	2.458E-08	1180/1053	552/730	0.681	0.591	
17	SLC6A4	rs2020939	6	GRIK2	rs2749074	0.5448	0.1012	1.44 (1.26:1.65)	8.982E-08	812/677	920/1106	0.469	0.380	

Linear models are presented in A-D and results from MDR analysis in E. All top interactions of each method are LD filtered (r^2^ < 0.2).

Abbreviations:

AP, attributable proportion due to interaction; Permuted P, permuted P value corrected for multiple comparisons; 00, 01, 10 and 11 denotes risk (1) or no risk (0) genotype for marker 1 and marker 2; OR, odds ratio estimates for the interaction term in the multiplicative method; P values of the multiplicative method are derived for the interaction term; AUC, area under the curve metrics derived from the ROC analysis (metrics of diagnose classifier for a particular set of par-wise markers); For the MDR method OR’s are calculated using the number of cases/controls in high and low risk groups as defined in the MDR analysis; Balanced accuracy, statistical metrics estimate how accurate individuals are classified into high versus low risk groups; The MDR P values are derived from test for significance of the OR estimates; TPR (true positive rate) and FPR (false positive rate) is the sensitivity and specificity metrics to test the diagnose classifier for a particular set of pair-wise markers; PAF, population attributable fraction, proportion of disease burden in the population due to exposure to genetic risk.

Notes: Asterisk (*) denotes that recoding of the preventive factors was made before measures of interaction in the additive scale were calculated. Recoding was done as follows:

*00 to 10, 01 to 11, 10 to 00 & 11 to 01.

**00 to 01, 01 to 11, 10 to 00 & 11 to 10.

***00 to 11, 01 to 10, 10 to 01 & 11 to 00.

For the additive model the strongest interaction effect, AP value of 0.72 (95% C.I., 0.42-1.02), was for markers in the genes *ARHGAP10* and *GRIK4*.

In the multiplicative interaction method (Table 1C-D) the strongest effect measure, for the interaction term β3, in the recessive model (OR_rec_ = 4.99, uncorrected P = 6.95E-5) was for markers in *GRIN2A* and *GRIN2B* genes and for the dominant model (OR_dom_ = 4.16, uncorrected P = 4.41E-5) for markers in *GRIK1* and *GRIK4* genes. Additionally, there was a marked interaction (OR_rec_ = 3.70) from the *ARHGAP10* gene in combination with the *SLC6A2* gene, and from the *HTR2A* gene in combination with the *COMT* gene (OR_dom_ = 3.05).

Using the model-free approach, there were no interaction events with large effect, OR < 1.51 (Table 1E). The accuracy for how classification could be predicted into high- versus low risk groups was relatively modest (top value = 0.55, permuted P = 0.14). The marginal difference in ratio of affected to unaffected individuals in high versus low risk groups explains the weak cross-validation metric (CVC) value of 4/10, confirming that the study population was obviously too small to identify shared genetic interacting loci with small effects with the MDR approach. Full details of result metrics of the top 10 interactions are provided Additional file 9: Table S4.

### Test for correlation between the different interaction methods

To find out to what extent the different algorithms identified pairs of markers of relevance to disease we performed a test for correlation between the three interactions methods. We computed the Pearson’s correlation coefficient (r) to test the covariance of interactions among the 0.5% region in one method against the entire dataset of another method. This test was used for combinations of all methods (Additional file 10: Figure S5A-L). Only weak correlations could be identified. The measures of correlation between the model-based methods and between the multiplicative dominant and MDR yielded r = 0.28 and r = 0.33 respectively. An even weaker correlation was found between the additive and MDR methods.

### Model fitting and population attributable fraction

For the 10 most significant interactions attributable fraction (PAF) scores, sensitivity and specificity scores or AUC values were calculated (Table 1A-E). No pairs of markers performed substantially better than any others. None of the reported top interactions on their own explained the diagnosis of MDD to large extent, confirming that two interacting genetic risk factors with major implications to MDD in the general population were not identified in the present study. This observation further support that the interacting genetic landscape is likely to be highly evolved and many interactions are underlying a diagnosis of MDD.

## Discussion

A key determinant to understand why people are at risk of complex disorders is the genetic architecture, i.e. number of risk loci, allele frequencies and interaction mechanism between genetic and non-genetic risk factors. In fact, the issue of missing heritability can most likely to a large extent be explained by interactions between risk factors.

Genetic risk for MDD is considered to partially be manifested from multiple genes which interact with each other [44]. We have performed an analysis looking at the genetic interacting landscape in candidate genes for MDD testing three different methods, each one designed to detect plausible statistical interaction effects. No significant interaction was identified. Without excluding the possibility that no genetic interaction with risk for MDD is present in these candidate genes, we speculate that our study sample is not large enough to permit identification of interacting genetic risk factors with small effect due to the high number of independent tests performed. We therefore hypothesize that the most significant interactions are enriched for interactions implicated in MDD-susceptibility from these candidate genes and thus provides indicative information for genetic risk for MDD (Table 1A-D).

Using the multiplicative interaction method, large effect sizes from the interaction term were reported from interactions in the glutamate system. Measured as the attributable proportion due to interaction (AP), the additive method identified pairs of markers with large effects. An important note is however that estimates of AP do not make any inference for how many exposures are present in the identified sufficient cause. Nor do they describe the number of sufficient causes that they are involved in. Hence, the AP value should not be interpreted as the proportion of risk to MDD that is explained by this interaction but the excess risk for MDD among individuals with both risk factors which are due to their interaction.

In the analysis using the model-free approach, the interaction with the best classifier value (balanced accuracy) was not repeatedly validated. The poor consistency of identified interactions is mirrored by the modest OR estimates and indicates the presence of many genetic factors that interact, each with only marginal risk contribution to MDD. Since we observed that the same markers were involved in several different 2-way models (Table 1) we employed a 3-way analysis for an additional subset of markers using the MDR approach. We found (Additional file 2: Supplementary Materials) that the top interaction was not consistently validated (3/10) and, without precluding the possibility that no synergy effects with relevance to MDD exist in these candidate genes, conclude that the curse of higher order of dimensions attenuates the power to predict an interaction in a sparse dataset.

From the results of the analysis of sensitivity and specificity scores, we conclude that none of the top interactions are on their own sufficient to explain why people develop MDD (Table 1A-E). Previous studies [45] indicate that the best disease classifiers are not necessarily found for interactions with low P values. Due to computational reasons, we have not searched our dataset for interactions with large classifier scores among less significant interaction events. Our interpretation is that the disease classifier scoring, along with the results from many separate interactions with small effect suggests that there is an extensive network of combined risk factors that underlies risk to MDD from these candidate genes. Detecting gene-gene interactions with relevance to risk of disorder can be more complicated and hard to interpret than expected, as theoretically, a certain gene could be protective in combination with one gene but risk conferring in combination with another gene. Following the same discussion, it is therefore unknown whether the expected effect size of interactions will be substantially larger than previously found in the single marker analyzes or whether the effect size will only be marginally larger. Despite the absence of significant interactions after correction for multiple testing, we advocate that the present study still holds crucial information that merits further attention. Firstly, the approach to include markers without LD filtering and with a limited restriction for pruning markers for predicted interaction effect provided insight into the interacting landscape in these 63 candidate genes. None of the top 10 interactions in any method and only a minimal proportion of the interactions in the top 0.5% region contained markers predicted to have interaction effects. However, in respect of the two logistic regression methods one of the pair-wise markers was in fact selected based on these two criteria and the observation in this case refers to interactions with both markers selected based on predicted effects. This is of significant interest since neither of the two a priori approaches we employed to select markers with predicted interactions effects seems to be optimal. This is a great challenge for future study designs if significantly interacting markers should be identified.

Secondly, to our knowledge none of the present top interactions have previously been reported to interact in risk for MDD. We performed a literature search on PubMed and the Genetic Association Database (http://geneticassociationdb.nih.gov/) and found relatively few gene-gene interaction analyses have been conducted for MDD. There are reports of interaction between *BDNF* and *NTRK2* and the 5-HTTLRP region in the *SLC6A4* gene [46,47]. In the present study, no interaction involving these three genes (in total 98 markers including the *BDNF* Val66Met polymorphism) were among the 0.5% most significant interactions in any of the methods. Thirdly, for the model-based approaches we had expected to find the largest effects in groups with few individuals. However, our preliminary results indicate that the genetic risk, in these candidate genes, is shared by many and confers risk with small effect.

Given the limited number of genotypes in the STAR*D study that were available at the time when this study was initiated, in comparison to marker coverage in GAIN, greatly reduced the possibility to produce identical markers between the STAR*D and the GAIN samples. This situation has implications for our interpretation of genetic heterogeneity between the two study populations. We made the conclusion based on the nominal P value from single marker analysis and cannot exclude the possibility that our decision would have been different if a much larger study populations would have been available. Nevertheless, the study sample was predetermined and given the circumstances with low power we conclude that for the given marker coverage the two samples were heterogeneous in the genetic risk to MDD. We stress that even though we presumed that the two study populations are different in the genetic risk to MDD based on the single-marker analysis we cannot exclude that the interaction effects might be shared.

Considering the region harboring the top 0.5% interactions we observed correlation between P values from interactions for the model-based methods, and between the multiplicative dominant and MDR methods. These correlations were however weak (r value close to zero, although significant). Additionally, only a proportion of the top 0.5% interactions were found among the top 0.5% interactions in another method (Additional file 10: Figure S5A-L). We, therefore, conclude that the different methods for testing interactions will not select the same pairs of markers as potential candidates to explain the risk of MDD which is not surprising given the different assumption behind the methods. These results are consistent with those from experimental genetic interaction studies in model systems which show that interactions corresponds to definitions of both additive and multiplicative models [48] which support our study design to search for interaction using different definitions.

The observation of negative AP estimates below −1.0 in our dataset was unexpected (Table 1A-B). AP is defined as the proportion of disease that is attributed due to interaction among those who are exposed to both risk factors [49,50]. It has been claimed that AP values can vary between +1 and −1 [51]. AP > 0 means excess risk, or positive interaction, and AP < 0 means negative interaction, or protection between the two risk factors [51,52]. It is therefore surprising that we frequently observed values below −1.0 in our dataset. It has previously been shown that measures of additive interactions calculated by substituting OR with RR in case–control studies may not be reliable estimates when the incidence of the disease is high, as the case for MDD where it is 15%,with inflation of the AP estimates as a consequence [52]. This cannot however be the only explanation for AP values below −1.0 as we have observed the same distribution in a dataset (data not shown) where we tested interaction between susceptibility SNPs for multiple sclerosis which has a prevalence less than ~0.2% [53]. By correlating AP values to several other measures (Additional file 8: Figure S4A-D) we demonstrate that AP values are apparently not normally distributed [50] and moreover that negative AP values were unexpectedly inflated at a low number of individuals in any risk group, as well as at large OR values for single exposures. Our conclusion is that AP can attain large negative values per se. A closer look at the definition of AP reveals the reason; the denominator equals the OR of the full model, which sometimes is small for pairs of SNPs that exhibit negative interaction. Thus, our interpretation is that negative values have a meaning for estimating protection although it is hard to interpret the magnitude of that protective effect. Following the line of arguments and discussion above we decided to report on the complete dataset but only comment on interaction with positive or synergistic interactions, i.e. positive AP values.

### Limitations

This study has several limitations. Firstly, the GAIN sample was underpowered for finding genetic interacting risk factors with small effect. To retain the best power of the remaining sample we did not test for interactions using any sub-categorization of MDD diagnosis or gender as this would have reduced power. This might reduce our chance to find genetic interacting risk factors for these separate categories. Because the present study was shown to have limited power for identifying genetic risk factors with small effects we designed the interaction analysis without partitioning the GAIN sample into a testing and replication datasets. Our study should thus only be considered as an explorative study which needs further confirmation. Secondly, due to the lack of prior evidence for superiority of any method to give priority to markers with a predicted major role in interactions effects [25,54] we reduced our chances of reporting a significant interaction through our massive screening approach.

## Conclusions

Our main objective of finding interacting genetic risk loci for MDD-susceptibility was not fruitful. Despite the absence of significant results we advocate that there are still interesting results in the present study. Firstly, with restriction to the 63 candidate genes our results further support the hypothesis that MDD has an ologogenetic background with an exceptionally evolved network of genetic interactions contributing small amount to the overall-risk of developing MDD. Secondly, our study design to test all markers without giving priority to a sub-sample of markers based on predicted interactions gave vital information. None of the most significant interactions contains markers based on main effect from single marker analysis or based on prediction using an a priori algorithm. Moreover, we also conclude that there is a little overlap in interacting markers identified in the different algorithms used. This might argue that none of the algorithms are adequate on their own. Thus, our results motivate that both model-based and model-free methods should be applied if interacting markers for complex traits shall be identified.

Identification of such interactions may help us to prioritize which biological processes that should be studied further in order to better understand MDD pathogenesis.

## Competing interests

The authors declare that they have no competing interests.

## Authors’ contributions

ML, IK & OH; responsible for (i) calculations and statistical analyses (ii) analysis plan and study design (iii) development of logistic regression model (iv) interpretation and conclusion of results and (v) writing of manuscript. PA & JHM; responsible for (i) the data mining machine learning (DMM) approach (ii) and interpretation and conclusions of results. HK; responsible for development of the GEIRA software. DU; responsible for coding the software calculating interaction using a logistic regression model. DC, HM, JAR & FJM; responsible for (i) selection of candidate genes, marker selection, SNP-genotyping and collection of the STAR*D sample and (ii) interpretation and conclusion of results. All authors read and approved the final manuscript.

## Supplementary Material

Additional file 1: Figure S1 Flow chart of analytic strategy and summary findings. A brief overview of our incremental strategy for finding shared common genetic risk factors using an exploratory study population (STAR*D) and a replication sample (GAIN) and finally to explore the genetic interacting landscape in MDD. Number of SNPs, analysis method and the main findings are presented at each stage. All P values related to single marker analysis in the figure are uncorrected for multiple comparisons. Click here for file

Additional file 2 Supplementary Materials. Click here for file

Additional file 3: Table S1 List of 68 candidate genes. Gene names, genomic size and functions were generated using the UCSC and KEGG databases (http://www.genome.ucsc.edu/, http://www.genome.jp/kegg/). Genes are listed according to proposed hypotheses for involvement in regulation of MDD-susceptibility. Numbers of genotyped SNPs that have passed the quality control filtering for each gene are also presented. Markers maps within 10 kb of each candidate gene according to the NCBI36/hg18. Click here for file

Additional file 4: Table S2 Power calculations. A: Input parameters for power calculation and resulting mean power estimates in each separate study. Power estimates were calculated under the significance level of 0.05. Asterisk (*) indicates the power estimates using Bonferroni’s corrected P value for significance in each study population; 0.05/654 (STAR*D) and 0.05/2,896 (1,516 + 1,380) (GAIN). B: Output trend statistics under each separate parameter value settings (corresponding to a significance level of P < 0.05). Click here for file

Additional file 5: Figure S2 -Log_10_ P values plotted for the single markers tested in the STAR*D and GAIN samples. The 68 candidate genes are arranged in a relative scale along chromosome 1–22 and chromosome X. We performed a bioinformatic search and found no overlap between nominally associated markers in the STAR*D or GAIN samples with literature findings in the NHGRI GWAS catalog for risk to MDD. Nor were marker replicated in either the discovery or replication analysis in the mega-analysis of genome-wide association studies for major depressive disorder (Major Depressive Disorder Working Group of the Psychiatric GWAS Consortium Mol Psychiatry. 2013. doi:10.1038/mp.2012.21). However, two nominally associated markers in the GAIN sample were located in two genes (GRIA1 and GRIK3) which were found to be reported for risk to schizophrenia in NHGRI GWAS catalog. A: −Log_10_ of the P values are plotted for 654 genotyped markers in the STAR*D sample. The 10 nominally associated markers that were identical with markers genotyped in the GAIN dataset and included in the meta-analysis are highlighted in red. Threshold values for the nominal P value of 0.05 and Bonferroni’s corrected P value (7.6E-5) are illustrated. B: −Log_10_ of the P values are plotted for 1,516 genotyped (black) and 1,380 imputed (blue) markers in the GAIN sample. The 10 nominally associated markers that were identical with nominally associated markers in the STAR*D dataset and included in the meta-analysis are highlighted in red. Threshold values for the nominal P value of 0.05 and Bonferroni’s corrected P value (1.7E-5) are illustrated. C: −Log_10_ of the P values are plotted for the 10 markers tested in the meta-analysis. Click here for file

Additional file 6: Table S3 Nominally associated markers identified from single marker analysis in the STAR*D and GAIN samples. A: Results of single marker association in STAR*D. B: Results of single marker association in GAIN. C: Results of single marker association in GAIN (imputed markers). Click here for file

Additional file 7: Figure S3 Results of SNP-SNP interaction analyses. -Log_10_ P or balanced accuracy values of the interaction tests are plotted against the rank of the test in each method. Column charts depicts (i) number of interactions at P < 0.05 (ii) number of interactions in top 0.5% region and (iii) the number of interactions with predicted effects (main effects, P < 0.05, or using an a priori algorithm) in the total dataset. Number of unique markers for each method in top 0.5% region is also illustrated. A and B: P values are plotted, which are derived from significance testing from estimates of the attributable proportion due to interaction (AP) in the additive method and from the interaction term for the multiplicative method (dominant model in black and recessive model in blue). Threshold for significance after a 1,000-fold permutation analysis accounting for number of comparisons (5% significance level) are illustrated as well as the cut-off values representing nominal P value and the top 0.5% of all interactions that were tested in each method. QQ-plots (with 95% C.I.) are illustrated for additive and multiplicative method. In the QQ-plot of the additive dominant and recessive models negative estimates of AP values illustrated with green dots. C: Interaction results of ~6.1 million ranked interactions in the MDR analysis plotted against balanced accuracy value. Threshold after a 1,000-fold permutation analysis is illustrated as well as the threshold line to define the top 0.5% of all interactions. In a subsequent analysis we observed that all 63 candidate genes are represented in the top 0.5% region in all tested methods (data not shown). Moreover, the interactions present among the 0.5% most significant ones were not restricted to a small group of markers. Click here for file

Additional file 8: Figure S4 Evaluation of AP values. A: Histogram of AP values. B: The lowest number of individuals in any exposure group, i.e. A0B0, A0B1, A1B0 or A1B1 (see Methods for definition of exposure groups), plotted against AP values. C: The lower of the two OR values from single markers (in any of the A0B1 or A1B0 groups) plotted against AP values. D: The higher of the two OR values from single markers (in any of the A0B1 or A1B0 groups) plotted against AP values. Click here for file

Additional file 9: Table S4 Detailed results for the 10 most nominally significant interactions. Linear models are presented in A-D and results from MDR analysis in E. All top 10 interactions of each method are LD filtered (r^2^ < 0.2). Abbreviations: AP, attributable proportion due to interaction; Permuted P, permuted P value, corrected for multiple comparisons; 00, 01, 10 and 11 denotes risk (1) or no risk (0) genotype for marker 1 and marker 2; OR, odds ratio estimates for the interaction term in the multiplicative method; P values of the multiplicative method are derived for the interaction term; AUC, area under the curve metrics derived from the ROC analysis (metrics for the diagnose classifier for a particular set of par-wise markers); For the MDR method OR's are calculated using the number of cases/controls in high and low risk groups as defined in the MDR analysis; Balanced accuracy, statistical metrics denotes how accurately individuals are classified into high versus low risk groups; The MDR P values are derived from test for significance of the OR estimates; TPR (true positive rate) and FPR (false positive rate) is the sensitivity and specificity metrics to test the diagnose classifier for a particular set of pair-wise markers; PAF, population attributable fraction, proportion of disease burden in the population due to exposure to genetic risk. For each MDR risk group (high risk and low risk) are the number of cases/controls and genotype combinations displayed (-2 denote missing genotype). Notes: Asterisk (*) denotes that recoding of the preventive factors was made before measures of interaction in the additive scale were calculated. Recoding was done as follows: * 00 to 10, 01 to11, 10 to 00 and 11 to 01. ** 00 to 01, 01 to 11, 10 to 00 and 11 to 10. *** 00 to 11, 01 to 10, 10 to 01 and 11 to 00. Click here for file

Additional file 10: Figure S5 Overlap of interaction results between different methods. A-L: Comparison of shared interactions between the top 0.5% regions (red dots) of one particular interaction method with the complete dataset (black dotted line) of another. The insert figures illustrate the correlation for shared interactions between two analysis methods. The gray dashed lines depict the highest value of the two methods and the 0.5% threshold respectively. Pearson’s correlation coefficient (r) was computed for the shared interactions in the top 0.5% regions. The r statistics and P values for each correlation analysis are depicted. Of note, as shown in the insert figures there was an absence of observations for shared interactions of the absolute top regions (very few observations are observed close to the gray dotted lines ‘highest value’). Thus, the remaining shared interactions yield a marked correlation statistics masking the low correlation of the interactions in the absolute top regions. Of note, as the interaction analysis in the MDR approach allowed to test all possible pair-wise SNP combinations which was not practically feasible in the additive or multiplicative models the comparison between the MDR versus logistic regression models are not fully illustrative. Click here for file
