# Supplementary material for: The genetic interacting landscape of 63 candidate genes in Major Depressive Disorder: an explorative study
Source: BioData Min. 2014 Sep 9;7:19. doi: 10.1186/1756-0381-7-19 (PMC4181757; doi:10.1186/1756-0381-7-19)
Supplement: Additional file 2 — Supplementary Materials. [file 1756-0381-7-19-S2.doc]

| **Table S1:** List of 68 candidate genes | | |  |  |  |
| --- | --- | --- | --- | --- | --- |
|  |  |  |  | **SNP/gene** | |
| **Hypothesis and gene-ID** | **Chr** | **Full gene name** | **Genomic size (bp)** | **STAR*D** | **GAIN** |
|  |  |  |  |  |  |
| **Dopamine hypothesis:** |  |  |  |  |  |
| TH | 11 | Tyrosine hydroxylase | 7877 | 3 | 6 |
| COMT | 22 | Catechol-o-methyltransferase | 27222 | 7 | 10 |
| MAOA | 23 | Monoamine-oxidase A | 90660 | 2 | 6 |
|  |  |  |  |  |  |
| **Adrenergic hypothesis:** | |  |  |  |  |
| ADRA2A | 10 | Alpha-2A adrenergic receptor | 3650 | 4 | 2 |
| ADRA2C | 4 | Alpha-2C adrenergic receptor | 2144 | 2 | 2 |
| DBH | 9 | Dopamine beta-hydroxylase | 22982 | 1 | 18 |
| SLC6A2 | 16 | Norepinephrine transporter | 47679 | 8 | 27 |
|  |  |  |  |  |  |
| **Serotonin hypothesis:** |  |  |  |  |  |
| SLC6A4 | 17 | Serotonin transporter | 37810 | 4 | 6 |
| TPH1 | 11 | Tryptophane hydroxylase 1 | 19772 | 8 | 7 |
| TPH2 | 12 | Tryptophane hydroxylase 2 | 93596 | 8 | 22 |
| HTR1A | 5 | 5-hydroxytryptamine receptor 1A | 1269 | 2 | 2 |
| HTR1B | 6 | 5-hydroxytryptamine receptor 1B | 1173 | 1 | 3 |
| HTR1D | 1 | 5-hydroxytryptamine receptor 1D | 2835 | 2 | 1 |
| HTR1E | 6 | 5-hydroxytryptamine receptor 1E | 78988 | 6 | 14 |
| HTR1F | 3 | 5-hydroxytryptamine receptor 1F | 11194 | 1 | 2 |
| HTR2A | 13 | 5-hydroxytryptamine receptor 2A | 63482 | 15 | 42 |
| HTR2B | 2 | 5-hydroxytryptamine receptor 2B | 16870 | 1 | 4 |
| HTR2C | 23 | 5-hydroxytryptamine receptor 2C | 326074 | 1 | 11 |
| HTR3A | 11 | 5-hydroxytryptamine receptor 3A | 15125 | 2 | 10 |
| HTR3B | 11 | 5-hydroxytryptamine receptor 3B | 41695 | 7 | 10 |
| HTR3C | 3 | 5-hydroxytryptamine receptor 3C | 7627 | 3 | 4 |
| HTR3D | 3 | 5-hydroxytryptamine receptor 3D | 7628 | 4 | 8 |
| HTR3E | 3 | 5-hydroxytryptamine receptor 3E | 6817 | 2 | 4 |
| HTR4 | 5 | 5-hydroxytryptamine receptor 4 | 172623 | 10 | 36 |
| HTR5A | 7 | 5-hydroxytryptamine receptor 5A | 14914 | 2 | 7 |
| HTR6 | 1 | 5-hydroxytryptamine receptor 6 | 14276 | 2 | 5 |
| HTR7 | 10 | 5-hydroxytryptamine receptor 7 | 117096 | 8 | 10 |
|  |  |  |  |  |  |
| **Glutamate hypothesis:** |  |  |  |  |  |
| GRIA1 | 5 | Glutamate receptor (AMPA) | 323346 | 28 | 87 |
| GRIA2 | 4 | Glutamate receptor (AMPA) | 140411 | 6 | 15 |
| GRIA3 | 23 | Glutamate receptor (AMPA) | 20436 | 38 | 7 |
| GRIA4 | 11 | Glutamate receptor (AMPA) | 372020 | 22 | 46 |
| GRIN1 | 9 | NMDA receptor subunit 1 | 29600 | 4 | 0 |
| GRIN2A | 16 | NMDA receptor subunit 2A | 419745 | 39 | 139 |
| GRIN2B | 12 | NMDA receptor subunit 2B | 418613 | 73 | 170 |
| GRIN2C | 17 | NMDA receptor subunit 2C | 16796 | 2 | 5 |
| GRIN2D | 19 | NMDA receptor subunit 2D | 50057 | 3 | 6 |
| GRIN2A | 9 | NMDA receptor subunit 3A | 169228 | 10 | 32 |
| GRIK1 | 21 | Glutamate receptor (Kainate) | 358538 | 44 | 71 |
| GRIK2 | 6 | Glutamate receptor (Kainate) | 671026 | 52 | 134 |
| GRIK3 | 1 | Glutamate receptor (Kainate) | 229616 | 8 | 35 |
| GRIK4 | 11 | Glutamate receptor (Kainate) | 421825 | 50 | 97 |
| GRIK5 | 19 | Glutamate receptor (Kainate) | 67485 | 2 | 2 |
| SLC1A1 | 9 | Solute carrier family 1 (glutamate transporter) | 97026 | 15 | 58 |
|  |  |  |  |  |  |
| **Other signaling pathways:** | |  |  |  |  |
| PPP1R1B | 17 | Protein phosphatase 1 | 8126 | 1 | 1 |
| CREB1 | 2 | cAMP responsive element binding protein 1 | 68885 | 4 | 3 |
| MAPK1 | 22 | Mitogen-activated protein kinase 1 | 98487 | 7 | 6 |
| GSK3B | 3 | Glycogen synthase kinase 3 beta | 266968 | 2 | 12 |
| CAMK1 | 3 | Calcium/calmodulin-dependent protein kinase 1 | 5634 | 1 | 2 |
| PPP3R2 | 9 | Protein phosphatase 3 regulatory subunit 3 | 3387 | 1 | 15 |
| ARHGAP10 | 4 | Rho-GTPase activating protein 10 | 190939 | 1 | 43 |
| GNB3 | 12 | Guanine nucleotide binding protein | 6525 | 1 | 7 |
| NR1I2 | 3 | Orphan nuclear receptor | 7410 | 1 | 4 |
|  |  |  |  |  |  |
| **Neurotrophin hypothesis:** | |  |  |  |  |
| BDNF | 11 | Brain-derived neurotrophic factor | 46712 | 4 | 10 |
| NTRK2 | 9 | Trk-B, Neurotrophic tyrosine kinase receptor 2 | 355040 | 36 | 17 |
| BCL2 | 18 | B-cell lymphoma protein 2 | 196079 | 27 | 4 |
| BAG1 | 9 | BCL2-associated athanogene | 12292 | 3 | 1 |
|  |  |  |  |  |  |
| **Hypothalamic-Pituitary-Adrenal-axis hypothesis:** | | |  |  |  |
| FKBP5 | 6 | FK506 binding protein 5 | 155031 | 2 | 15 |
| NR3C2 | 4 | Mineralcorticoid receptor | 363729 | 33 | 80 |
|  |  |  |  |  |  |
| **DNA-repair, Epigenetic & Transcriptional modifications:** | | |  |  |  |
| OGG1 | 3 | 8-oxoguanine DNA glycosylase | 17638 | 1 | 0 |
| GRWD1 | 19 | Glutamate-rich WD repeat containing 1 | 8093 | 1 | 1 |
| RPP30 | 10 | Ribonuclease P | 36839 | 1 | 6 |
| RNF20 | 9 | Ring finger protein 20 | 29494 | 1 | 10 |
| KDELR1 | 19 | ER lumen protein retaining receptor 1 | 8984 | 1 | 3 |
|  |  |  |  |  |  |
| **Cell cycle regulation:** |  |  |  |  |  |
| FBXO38 | 5 | F-box protein 38 | 58854 | 1 | 6 |
| NBL1 | 1 | Neuroblastoma suppression of tumorigenicity 1 | 14697 | 1 | 1 |
|  |  |  |  |  |  |
| **Cell adhesion, Cell signaling:** | |  |  |  |  |
| LAMA4 | 6 | Laminin alpha-4 | 145850 | 7 | 50 |
| TSPAN9 | 12 | Tetraspanin 9 | 209174 | 4 | 35 |
|  |  |  |  |  |  |
| **Electrochemical gradient:** | |  |  |  |  |
| ATP1A3 | 19 | Sodium/Potassium -ATPase alpha 3 | 27649 | 1 | 1 |
