# Supplementary material for: The genetic interacting landscape of 63 candidate genes in Major Depressive Disorder: an explorative study
Source: BioData Min. 2014 Sep 9;7:19. doi: 10.1186/1756-0381-7-19 (PMC4181757; doi:10.1186/1756-0381-7-19)
Supplement: Additional file 3: Table S1 — List of 68 candidate genes. Gene names, genomic size and functions were generated using the UCSC and KEGG databases (http://www.genome.ucsc.edu/, http://www.genome.jp/kegg/). Genes are listed according to proposed hypotheses for involvement in regulation of MDD-susceptibility. Numbers of genotyped SNPs that have passed the quality control filtering for each gene are also presented. Markers maps within 10 kb of each candidate gene according to the NCBI36/hg18. [file 1756-0381-7-19-S3.doc]

| **Table S2:** Power calculations | | |
| --- | --- | --- |
|  |  |  |
| **A:** Estimated model parameters and power calculations | | |
| **Input parameters** | **STAR*D** | **GAIN** |
| Number of affected individuals | 1240 | 1732 |
| Number of unaffected individuals | 630 | 1783 |
| Test (weights; X0=0, X1=1, X2=2) | Linear trend | Linear trend |
| Plot type | Relative risk | Relative risk |
| Significance level | 0.05 | 0.05 |
| Mode | Additive | Additive |
| R1 (relative risk heterozygout geneotype) | 1.1-1.3 | 1.1-1.3 |
| R2 (relative risk homozygout geneotype) | 1.35-1.7 | 1.35-1.7 |
| Pd (allele frequency disease causing allele) | 0.05-0.40 | 0.05-0.40 |
| P1 (allele frequency marker allele) | 0.05-0.40 | 0.05-0.40 |
| K (prevalence disorder) | 0.10-0.15 | 0.10-0.15 |
| LD measure (r2) between marker & disease allele) | 0.8 | 0.8 |
| Theta (diagnostic errors probability for cases) | 0.01-0.05 | 0.01-0.05 |
| Phi (diagnostic error probability for controls) | 0.01-0.05 | 0.01-0.05 |
| **Calculated mean power:** | 32% (1.4%)* | 53% (4.3%)* |

| **B:** Linear trend statistics | | | |  |  |  |  |  |  |  |  |  |  |  |  |  |  |
| --- | --- | --- | --- | --- | --- | --- | --- | --- | --- | --- | --- | --- | --- | --- | --- | --- | --- |
|  | **STAR*D** | | | | | | | |  | **GAIN** | | | | | | | |
|  | **Power** | **R1** | **R2** | **P1** | **Pd** | **K** | **Theta** | **Phi** |  | **Power** | **R1** | **R2** | **P1** | **Pd** | **K** | **Theta** | **Phi** |
| **Mean** | 0.32 |  |  |  |  |  |  |  |  | 0.53 |  |  |  |  |  |  |  |
| **Min** | 0.05 | 1.10 | 1.48 | 0.36 | 0.05 | 0.12 | 0.04 | 0.03 |  | 0.05 | 1.10 | 1.37 | 0.39 | 0.05 | 0.11 | 0.03 | 0.05 |
| **0.1** | 0.11 | 1.28 | 1.70 | 0.38 | 0.09 | 0.11 | 0.02 | 0.03 |  | 0.18 | 1.27 | 1.40 | 0.05 | 0.38 | 0.12 | 0.02 | 0.01 |
| **0.25** | 0.17 | 1.21 | 1.49 | 0.11 | 0.06 | 0.14 | 0.04 | 0.01 |  | 0.31 | 1.28 | 1.37 | 0.31 | 0.12 | 0.12 | 0.05 | 0.03 |
| **Median** | 0.28 | 1.28 | 1.64 | 0.11 | 0.08 | 0.15 | 0.02 | 0.04 |  | 0.52 | 1.23 | 1.45 | 0.12 | 0.18 | 0.13 | 0.04 | 0.04 |
| **0.75** | 0.44 | 1.26 | 1.37 | 0.32 | 0.39 | 0.13 | 0.01 | 0.03 |  | 0.75 | 1.20 | 1.37 | 0.35 | 0.37 | 0.11 | 0.05 | 0.02 |
| **0.9** | 0.58 | 1.26 | 1.40 | 0.38 | 0.37 | 0.13 | 0.02 | 0.03 |  | 0.89 | 1.20 | 1.39 | 0.40 | 0.40 | 0.13 | 0.02 | 0.02 |
| **Max** | 0.92 | 1.30 | 1.66 | 0.39 | 0.40 | 0.14 | 0.04 | 0.01 |  | 1 | 1.27 | 1.70 | 0.36 | 0.37 | 0.14 | 0.03 | 0.01 |
