# Supplementary figures and images for: The genetic interacting landscape of 63 candidate genes in Major Depressive Disorder: an explorative study
Source: BioData Min. 2014 Sep 9;7:19. doi: 10.1186/1756-0381-7-19 (PMC4181757; doi:10.1186/1756-0381-7-19)

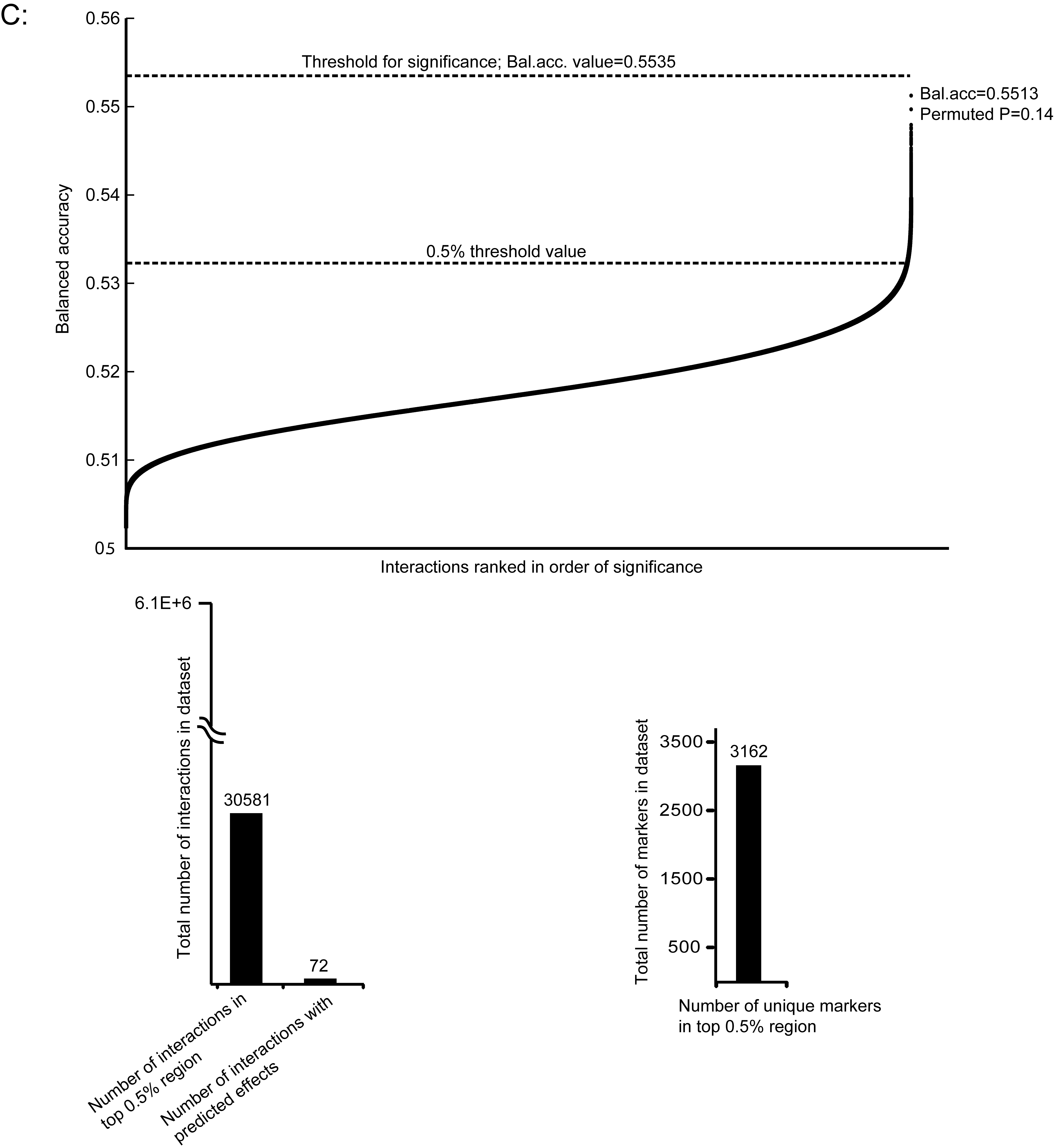

Supplement: Additional file 6: Table S3 — Nominally associated markers identified from single marker analysis in the STAR*D and GAIN samples. A: Results of single marker association in STAR*D. B: Results of single marker association in GAIN. C: Results of single marker association in GAIN (imputed markers). [file 1756-0381-7-19-S6.zip › 6384786221222972_add7_Figure_3C.tif]

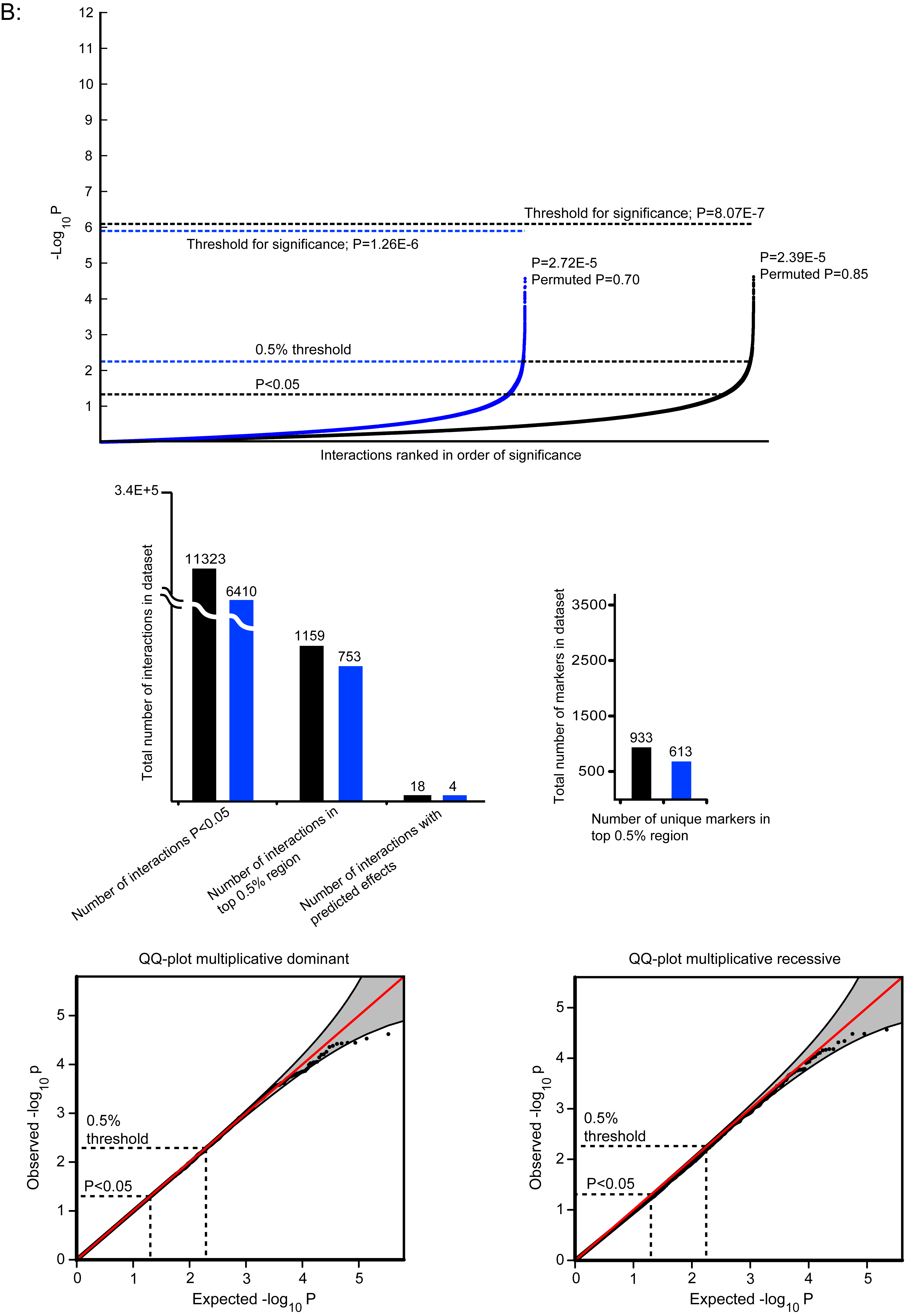

Supplement: Additional file 6: Table S3 — Nominally associated markers identified from single marker analysis in the STAR*D and GAIN samples. A: Results of single marker association in STAR*D. B: Results of single marker association in GAIN. C: Results of single marker association in GAIN (imputed markers). [file 1756-0381-7-19-S6.zip › 6384786221222972_add7_Figure_3B.tif]

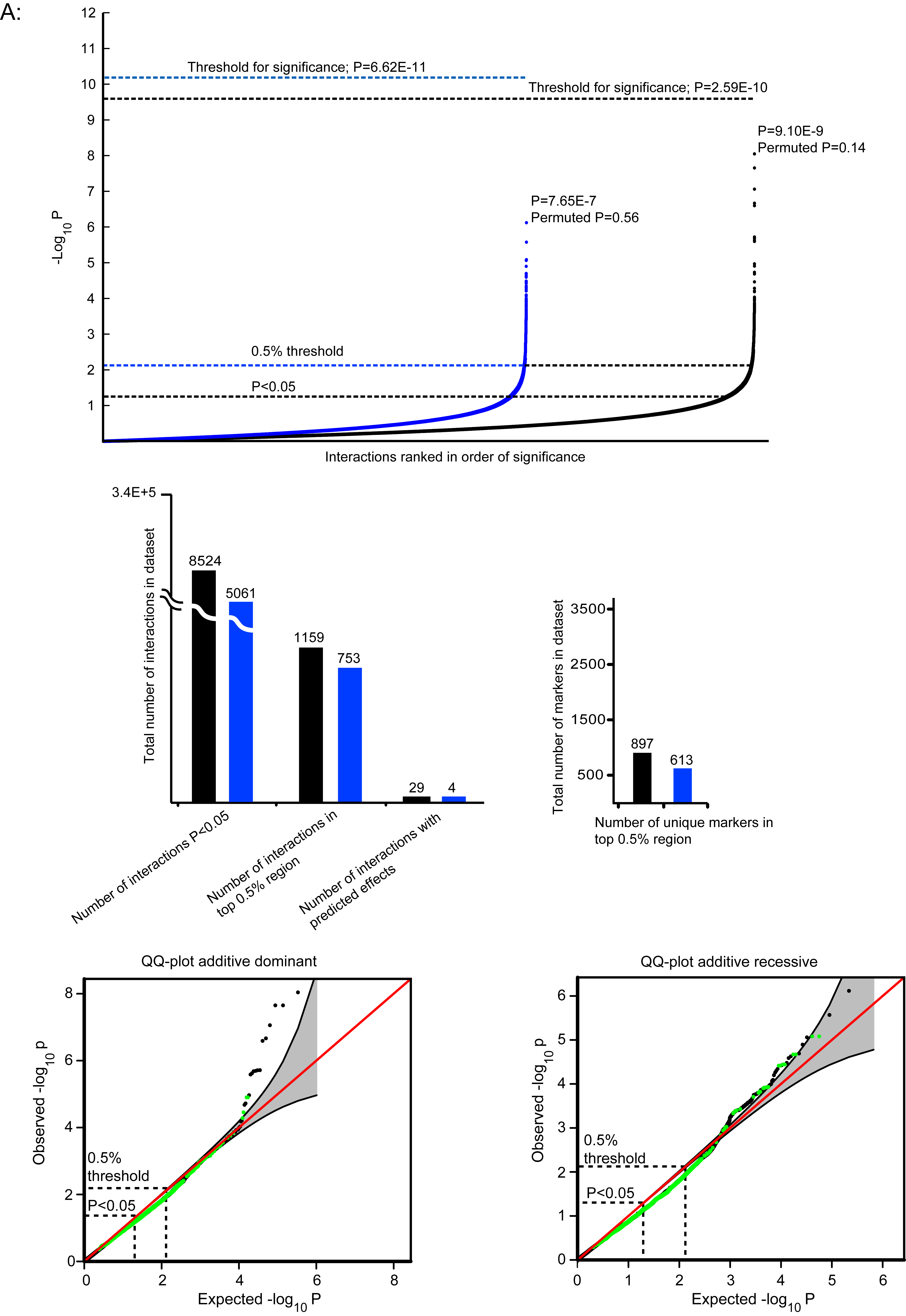

Supplement: Additional file 6: Table S3 — Nominally associated markers identified from single marker analysis in the STAR*D and GAIN samples. A: Results of single marker association in STAR*D. B: Results of single marker association in GAIN. C: Results of single marker association in GAIN (imputed markers). [file 1756-0381-7-19-S6.zip › 6384786221222972_add7_Figure_3A.tif]

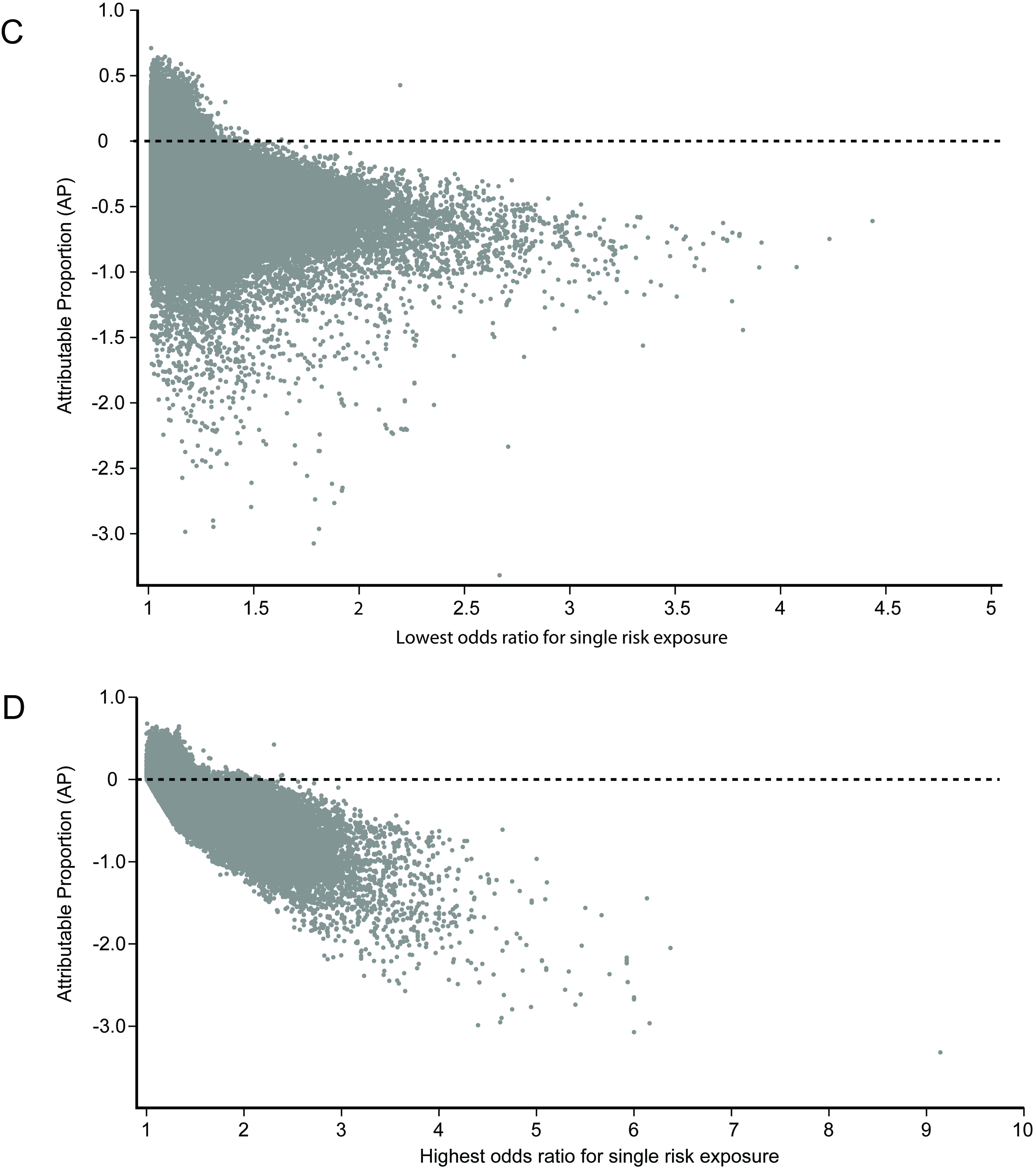

Supplement: Additional file 7: Figure S3 — Results of SNP-SNP interaction analyses. -Log10 P or balanced accuracy values of the interaction tests are plotted against the rank of the test in each method. Column charts depicts (i) number of interactions at P < 0.05 (ii) number of interactions in top 0.5% region and (iii) the number of interactions with predicted effects (main effects, P < 0.05, or using an a priori algorithm) in the total dataset. Number of unique markers for each method in top 0.5% region is also illustrated. A and B: P values are plotted, which are derived from significance testing from estimates of the attributable proportion due to interaction (AP) in the additive method and from the interaction term for the multiplicative method (dominant model in black and recessive model in blue). Threshold for significance after a 1,000-fold permutation analysis accounting for number of comparisons (5% significance level) are illustrated as well as the cut-off values representing nominal P value and the top 0.5% of all interactions that were tested in each method. QQ-plots (with 95% C.I.) are illustrated for additive and multiplicative method. In the QQ-plot of the additive dominant and recessive models negative estimates of AP values illustrated with green dots. C: Interaction results of ~6.1 million ranked interactions in the MDR analysis plotted against balanced accuracy value. Threshold after a 1,000-fold permutation analysis is illustrated as well as the threshold line to define the top 0.5% of all interactions. In a subsequent analysis we observed that all 63 candidate genes are represented in the top 0.5% region in all tested methods (data not shown). Moreover, the interactions present among the 0.5% most significant ones were not restricted to a small group of markers. [file 1756-0381-7-19-S7.zip › 6384786221222972_add8_Figure_4C-D.jpg]

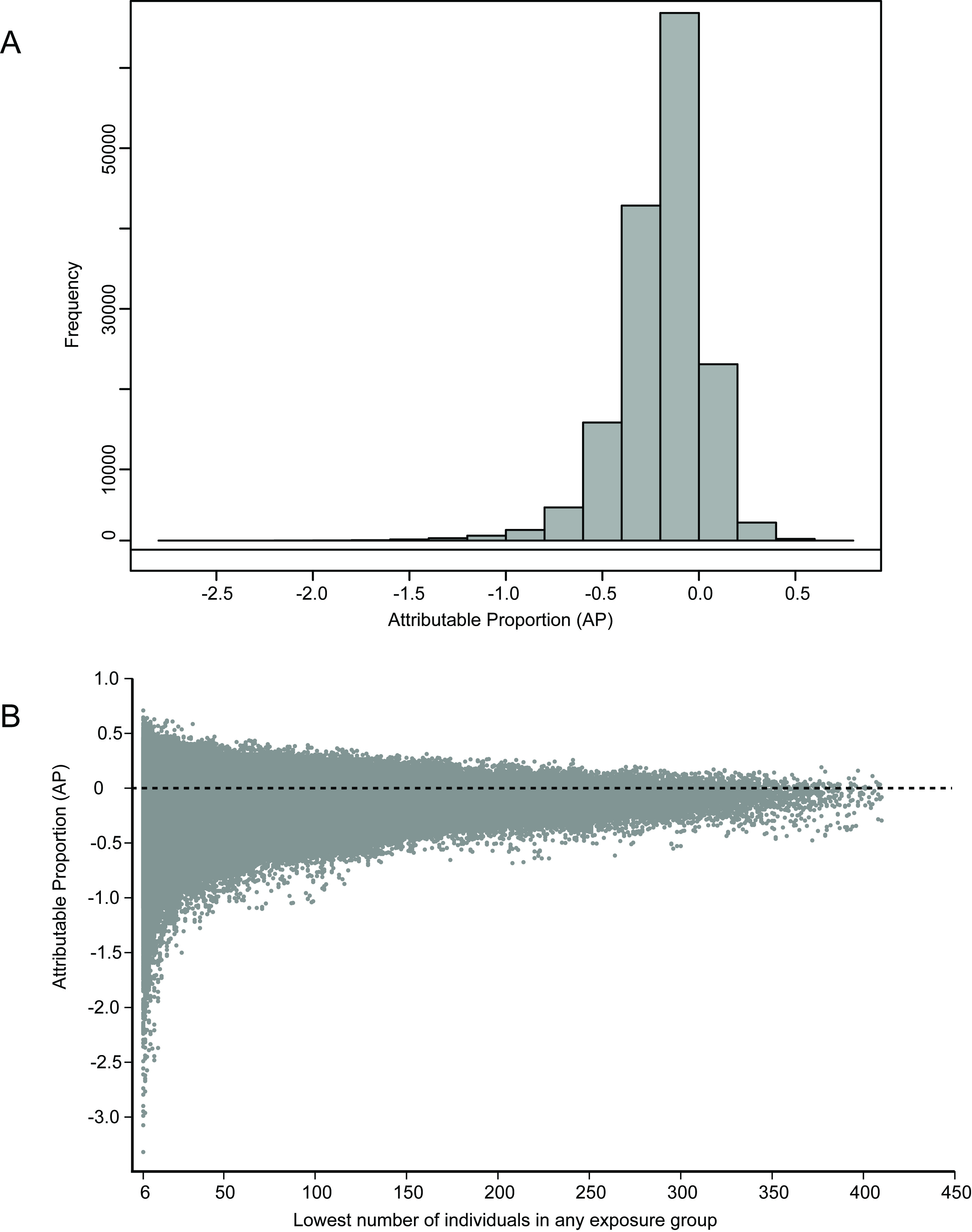

Supplement: Additional file 7: Figure S3 — Results of SNP-SNP interaction analyses. -Log10 P or balanced accuracy values of the interaction tests are plotted against the rank of the test in each method. Column charts depicts (i) number of interactions at P < 0.05 (ii) number of interactions in top 0.5% region and (iii) the number of interactions with predicted effects (main effects, P < 0.05, or using an a priori algorithm) in the total dataset. Number of unique markers for each method in top 0.5% region is also illustrated. A and B: P values are plotted, which are derived from significance testing from estimates of the attributable proportion due to interaction (AP) in the additive method and from the interaction term for the multiplicative method (dominant model in black and recessive model in blue). Threshold for significance after a 1,000-fold permutation analysis accounting for number of comparisons (5% significance level) are illustrated as well as the cut-off values representing nominal P value and the top 0.5% of all interactions that were tested in each method. QQ-plots (with 95% C.I.) are illustrated for additive and multiplicative method. In the QQ-plot of the additive dominant and recessive models negative estimates of AP values illustrated with green dots. C: Interaction results of ~6.1 million ranked interactions in the MDR analysis plotted against balanced accuracy value. Threshold after a 1,000-fold permutation analysis is illustrated as well as the threshold line to define the top 0.5% of all interactions. In a subsequent analysis we observed that all 63 candidate genes are represented in the top 0.5% region in all tested methods (data not shown). Moreover, the interactions present among the 0.5% most significant ones were not restricted to a small group of markers. [file 1756-0381-7-19-S7.zip › 6384786221222972_add8_Figure_4A-B.jpg]

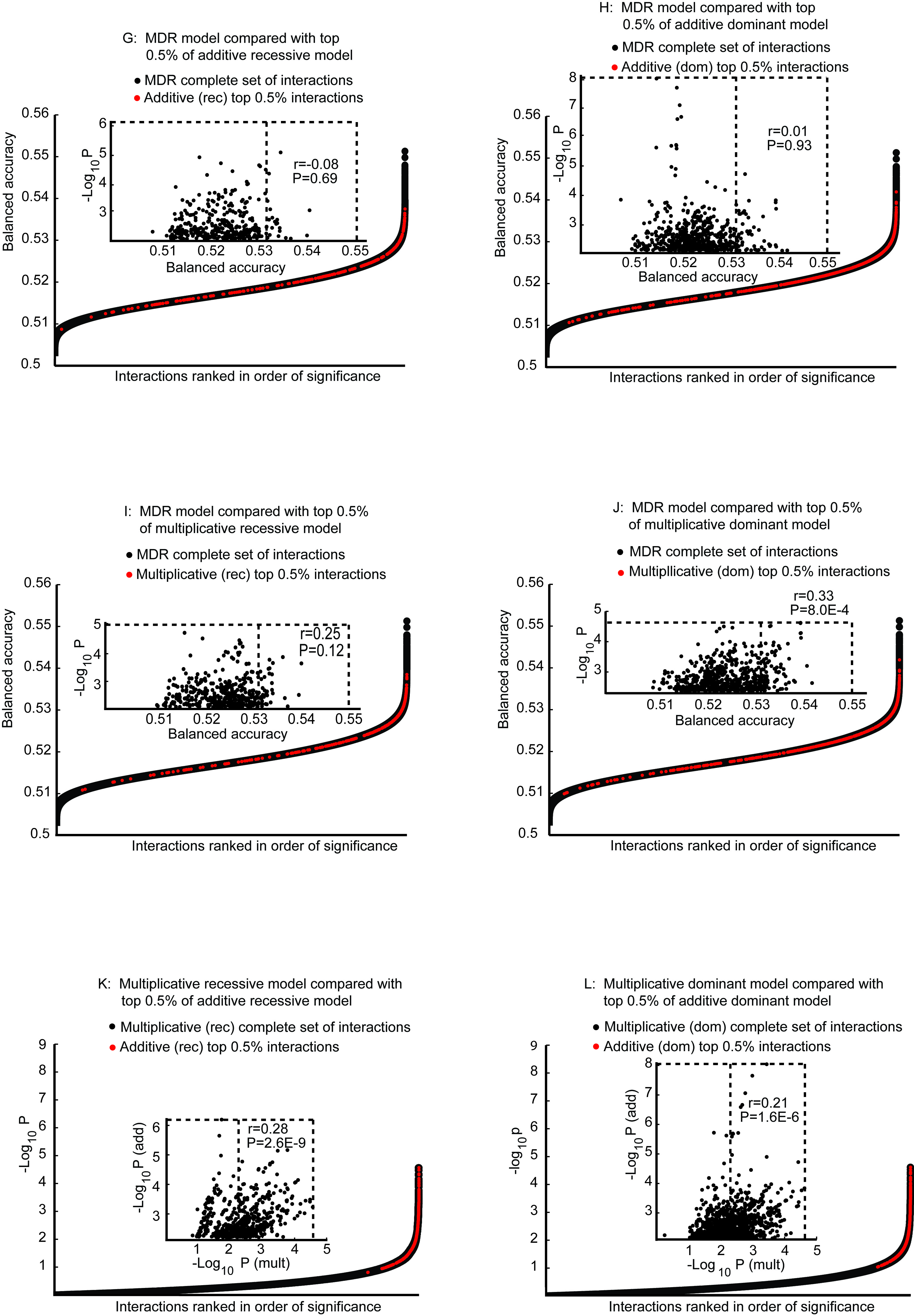

Supplement: Additional file 10: Figure S5 — Overlap of interaction results between different methods. A-L: Comparison of shared interactions between the top 0.5% regions (red dots) of one particular interaction method with the complete dataset (black dotted line) of another. The insert figures illustrate the correlation for shared interactions between two analysis methods. The gray dashed lines depict the highest value of the two methods and the 0.5% threshold respectively. Pearson’s correlation coefficient (r) was computed for the shared interactions in the top 0.5% regions. The r statistics and P values for each correlation analysis are depicted. Of note, as shown in the insert figures there was an absence of observations for shared interactions of the absolute top regions (very few observations are observed close to the gray dotted lines ‘highest value’). Thus, the remaining shared interactions yield a marked correlation statistics masking the low correlation of the interactions in the absolute top regions. Of note, as the interaction analysis in the MDR approach allowed to test all possible pair-wise SNP combinations which was not practically feasible in the additive or multiplicative models the comparison between the MDR versus logistic regression models are not fully illustrative. [file 1756-0381-7-19-S10.zip › 6384786221222972_add10_Figure_5G-L.jpg]

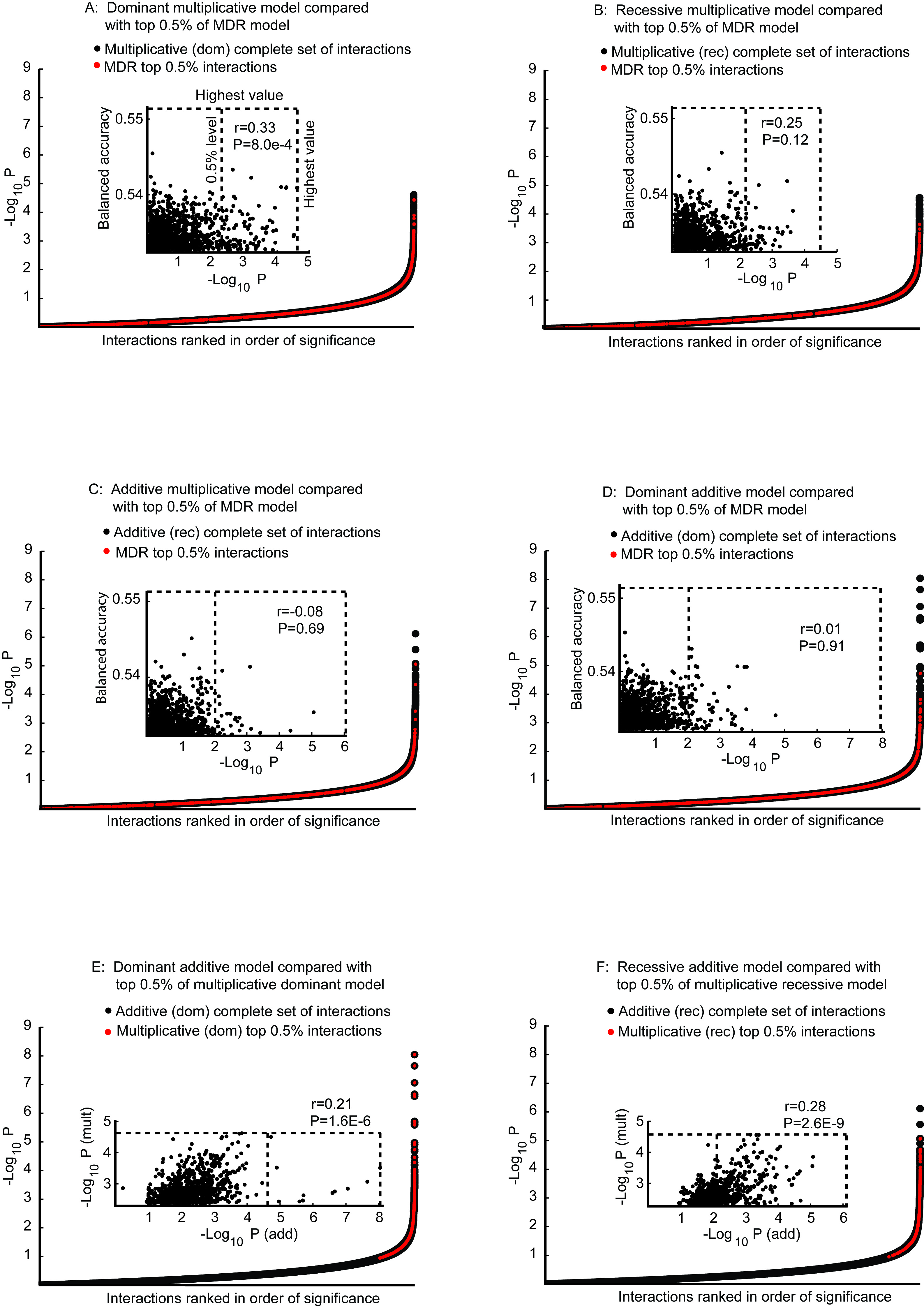

Supplement: Additional file 10: Figure S5 — Overlap of interaction results between different methods. A-L: Comparison of shared interactions between the top 0.5% regions (red dots) of one particular interaction method with the complete dataset (black dotted line) of another. The insert figures illustrate the correlation for shared interactions between two analysis methods. The gray dashed lines depict the highest value of the two methods and the 0.5% threshold respectively. Pearson’s correlation coefficient (r) was computed for the shared interactions in the top 0.5% regions. The r statistics and P values for each correlation analysis are depicted. Of note, as shown in the insert figures there was an absence of observations for shared interactions of the absolute top regions (very few observations are observed close to the gray dotted lines ‘highest value’). Thus, the remaining shared interactions yield a marked correlation statistics masking the low correlation of the interactions in the absolute top regions. Of note, as the interaction analysis in the MDR approach allowed to test all possible pair-wise SNP combinations which was not practically feasible in the additive or multiplicative models the comparison between the MDR versus logistic regression models are not fully illustrative. [file 1756-0381-7-19-S10.zip › 6384786221222972_add10_Figure_5A-F.jpg]
