# Supplementary material for: The genetic interacting landscape of 63 candidate genes in Major Depressive Disorder: an explorative study
Source: BioData Min. 2014 Sep 9;7:19. doi: 10.1186/1756-0381-7-19 (PMC4181757; doi:10.1186/1756-0381-7-19)
Supplement: Additional file 9: Table S4 — Detailed results for the 10 most nominally significant interactions. Linear models are presented in A-D and results from MDR analysis in E. All top 10 interactions of each method are LD filtered (r2 < 0.2). Abbreviations: AP, attributable proportion due to interaction; Permuted P, permuted P value, corrected for multiple comparisons; 00, 01, 10 and 11 denotes risk (1) or no risk (0) genotype for marker 1 and marker 2; OR, odds ratio estimates for the interaction term in the multiplicative method; P values of the multiplicative method are derived for the interaction term; AUC, area under the curve metrics derived from the ROC analysis (metrics for the diagnose classifier for a particular set of par-wise markers); For the MDR method OR's are calculated using the number of cases/controls in high and low risk groups as defined in the MDR analysis; Balanced accuracy, statistical metrics denotes how accurately individuals are classified into high versus low risk groups; The MDR P values are derived from test for significance of the OR estimates; TPR (true positive rate) and FPR (false positive rate) is the sensitivity and specificity metrics to test the diagnose classifier for a particular set of pair-wise markers; PAF, population attributable fraction, proportion of disease burden in the population due to exposure to genetic risk. For each MDR risk group (high risk and low risk) are the number of cases/controls and genotype combinations displayed (-2 denote missing genotype). Notes: Asterisk (*) denotes that recoding of the preventive factors was made before measures of interaction in the additive scale were calculated. Recoding was done as follows: * 00 to 10, 01 to11, 10 to 00 and 11 to 01. ** 00 to 01, 01 to 11, 10 to 00 and 11 to 10. *** 00 to 11, 01 to 10, 10 to 01 and 11 to 00. [file 1756-0381-7-19-S9.pdf]

**Table S4: Detailed results for the 10 most nominally significant interactions**

**A: Additive dominant**

| Marker 1 | | | | | | | Marker 2 | | | | | | | Interaction statistics | | | | | Cases/Controls with marker 1 and marker 2 conferring risk (1) or no-risk (0) | | | |
|---|---|---|---|---|---|---|---|---|---|---|---|---|---|---|---|---|---|---|---|---|---|---|
| Chr | Gene | Rs | P | OR (95% C.I.) | Minor/Major (risk) | PAF | Chr | Gene | Rs | P | OR (95% C.I.) | Minor/Major (risk) | PAF | AP (95% C.I.) | P | Permuted P | PAF | AUC | 00 | 01 | 10 | 11 |
| 4 | ARHGAP10 | rs9332471 | 0.03 | 0.81 (0.67:0.97) | C/T (C) | 0.133 | 4 | ARHGAP10 | rs6845865 | 0.41 | 0.95 (0.84:1.08) | C/T ( C) | 0.104 | 0.58 (0.39:0.78) | 9.10E-9 | 0.14 | 0.147 | 0.5153 | 1009/1073 | 169/174 | 475/500 | 78/33 |
| 6 | HTR1E | rs6922679 | 0.72 | 0.97 (0.83:1.14) | C/T ( C) | 0.122 | 16 | GRIN2A | rs17570500 | 0.90 | 1.01 (0.89:1.14) | C/A (A) | 0.104 | *0.71 (0.42:1.002) | 1.92E-6 | 0.57 | 0.142 | 0.5046 | 48/50 | 20/6 | 1332/1388 | 310/321 |
| 4 | ARHGAP10 | rs9332471 | 0.03 | 0.81 (0.67:0.97) | C/T (C) | 0.133 | 4 | ARHGAP10 | rs2306910 | 0.29 | 0.99 (0.76:1.09) | T/C (T) | 0.128 | 0.63 (0.37:0.89) | 2.40E-6 | 0.61 | 0.149 | 0.5136 | 1201/1273 | 190/182 | 233/238 | 35/12 |
| 4 | ARHGAP10 | rs9991394 | 0.07 | 0.87 (0.88:1.01) | C/T (C) | 0.121 | 4 | ARHGAP10 | rs12645249 | 0.09 | 0.84 (0.68:1.03) | T/C (T) | 0.135 | 0.59 (0.34:0.84) | 2.58E-6 | 0.63 | 0.146 | 0.5173 | 1162/1257 | 332/326 | 154/158 | 47/18 |
| 9 | GRIN3A | rs7873495 | 0.37 | 0.93 (0.79:1.10) | T/C (T) | 0.126 | 11 | GRIK4 | rs10892635 | 0.50 | 0.97 (0.87:1.07) | A/G (G) | 0.079 | **-1.08 (-1.56:-0.59) | 2.58E-7 | 0.90 | 0.139 | 0.5216 | 290/258 | 10/30 | 1308/1390 | 122/105 |
| 12 | GRIN2B | rs12371702 | 0.05 | 0.88 (0.76:0.99) | C/G ( C) | 0.113 | 9 | GRIN3A | rs13292935 | 0.77 | 0.97 (0.87:1.15) | T/C (T) | 0.126 | ***-0.92 (-1.35:-0.50) | 2.58E-8 | 0.95 | 0.146 | 0.5375 | 1003/1122 | 442/377 | 236/202 | 51/82 |
| 12 | GRIN2B | rs11832404 | 0.002 | 0.75 (0.62:0.90) | C/T ( C) | 0.132 | 4 | NR3C2 | rs982076 | 0.62 | 1.03 (0.93:1.15) | T/C ( C) | 0.086 | **-1.23 (-1.81:-0.65) | 2.58E-9 | 0.99 | 0.142 | 0.5266 | 257/182 | 10/24 | 1268/1367 | 92/86 |
| 12 | GRIN2B | rs11832404 | 0.002 | 0.75 (0.62:0.90) | C/T ( C) | 0.132 | 11 | GRIK4 | rs7928347 | 0.68 | 1.02 (0.92:1.37) | T/C ( C) | 0.087 | **-1.27 (-1.89:-0.66) | 2.58E-10 | 0.99 | 0.142 | 0.5300 | 255/185 | 7/18 | 1234/1332 | 87/78 |
| 4 | ARHGAP10 | rs9332471 | 0.03 | 0.81 (0.67:0.97) | C/T (C) | 0.133 | 9 | SLC1A1 | rs184204 | 0.21 | 0.90 (0.77:1.06) | T/C (T) | 0.125 | 0.53 (0.27:0.79) | 2.58E-11 | 0.99 | 0.145 | 0.5157 | 1212/1292 | 194/183 | 271/282 | 53/23 |
| 5 | GRIA1 | rs17114975 | 0.24 | 0.99 (0.75:1.07) | T/C (T) | 0.129 | 12 | GRIN2B | rs1012587 | 0.14 | 1.09 (0.97:1.22) | A/T (T) | 0.095 | *0.64 (0.33:0.95) | 2.58E-12 | 0.99 | 0.129 | 0.5084 | 83/79 | 24/8 | 1381/1448 | 241/245 |

**B: Additive recessive**

| Marker 1 | | | | | | | Marker 2 | | | | | | | Interaction statistics | | | | | Cases/Controls with marker 1 and marker 2 conferring risk (1) or no-risk (0) | | | |
|---|---|---|---|---|---|---|---|---|---|---|---|---|---|---|---|---|---|---|---|---|---|---|
| Chr | Gene | Rs | P | OR (95% C.I.) | Minor/Major (risk) | PAF | Chr | Gene | Rs | P | OR (95% C.I.) | Minor/Major (risk) | PAF | AP (95% C.I.) | P | Permuted P | PAF | AUC | 00 | 01 | 10 | 11 |
| 9 | RNF20 | rs16920473 | 0.02 | 0.85 (0.74:0.98) | T/C (C ) | 0.148 | 12 | GRIN2B | rs2216127 | 0.04 | 1.15 (1.01:1.31) | T/C (T) | 0.146 | 0.69 (0.42:0.97) | 7.65E-7 | 0.56 | 0.149 | 0.5238 | 377/446 | 1296/1305 | 12/14 | 46/14 |
| 4 | ARHGAP10 | rs4835456 | 0.02 | 1.18 (1.03:1.35) | A/T (A) | 0.146 | 11 | GRIK4 | rs4936540 | 0.25 | 0.95 (0.86:1.04) | G/A (A) | 0.129 | 0.72 (0.42:1.02) | 2.70E-6 | 0.72 | 0.148 | 0.5136 | 1105/1176 | 22/23 | 582/571 | 22/6 |
| 4 | ARHGAP10 | rs6824449 | 0.04 | 1.11 (1.003:1.22) | G/A (G) | 0.132 | 16 | SLC6A2 | rs192303 | 0.33 | 1.05 (0.95:1.16) | G/C (G) | 0.116 | ***-1.28 (-1.85:-0.72) | 8.30E-6 | 0.86 | 0.149 | 0.5319 | 1293/1414 | 208/159 | 184/144 | 16/32 |
| 16 | GRIN2A | rs4782040 | 0.36 | 1.05 (0.95:1.16) | A/G (A) | 0.138 | 11 | GRIK4 | rs11218032 | 0.87 | 1.01 (0.91:1.13) | A/T (A) | 0.126 | ***-0.99 (-1.43:-0.55) | 8.73E-6 | 0.87 | 0.149 | 0.5211 | 1244/1306 | 122/126 | 250/207 | 9/32 |
| 12 | GRIN2B | rs3764030 | 0.13 | 1.10 (0.97:1.24) | T/C (T) | 0.144 | 5 | HTR4 | rs6865654 | 0.24 | 1.06 (0.96:1.17) | T/C (T) | 0.129 | 0.68 (0.37:0.99) | 1.27E-5 | 0.91 | 0.148 | 0.5063 | 1363/1423 | 47/48 | 231/235 | 22/7 |
| 11 | GRIK4 | rs7939968 | 0.02 | 1.23 (1.02:1.24) | A/T (A) | 0.130 | 4 | NR3C2 | rs12641471 | 0.28 | 1.06 (0.96:1.16) | A/C (A) | 0.120 | 0.52 (0.28:0.76) | 2.03E-5 | 0.94 | 0.144 | 0.5170 | 1088/1165 | 192/186 | 275/292 | 69/32 |
| 4 | ARHGAP10 | rs6824449 | 0.04 | 1.11 (1.003:1.22) | G/A (G) | 0.132 | 16 | GRIN2A | rs11640235 | 0.64 | 1.03 (0.92:1.14) | G/C (G) | 0.140 | ***-1.01 (-1.56:-0.55) | 3.60E-5 | 0.98 | 0.149 | 0.5204 | 1355/1435 | 210/163 | 105/108 | 8/25 |
| 11 | GRIK4 | rs7939968 | 0.02 | 1.23 (1.02:1.24) | A/T (A) | 0.130 | 4 | NR3C2 | rs4835508 | 0.18 | 1.07 (0.97:1.18) | T/C (T) | 0.119 | 0.51 (0.27:0.75) | 3.77E-5 | 0.98 | 0.143 | 0.5186 | 1029/1118 | 185/179 | 275/295 | 70/33 |
| 11 | GRIK4 | rs7939968 | 0.02 | 1.23 (1.02:1.24) | A/T (A) | 0.130 | 4 | ARHGAP10 | rs12641157 | 0.57 | 1.03 (0.93:1.15) | T/G (T) | 0.140 | 0.64 (0.33:0.94) | 5.05E-5 | 0.99 | 0.148 | 0.5178 | 1347/1444 | 260/229 | 93/95 | 26/8 |
| 3 | HTR3C | rs6766410 | 0.01 | 1.13 (1.03:1.25) | A/C (A) | 0.119 | 6 | LAMA4 | rs6913656 | 0.93 | 1.00 (0.91:1.11) | G/A (G) | 0.129 | ***-0.84 (-1.25:-0.43) | 5.48E-5 | 0.99 | 0.148 | 0.5216 | 1210/1279 | 243/207 | 217/218 | 21/49 |

C: Multiplicative dominant

| Marker 1 | | | | | | | Marker 2 | | | | | | | Interaction statistics | | | | | | | | |
|---|---|---|---|---|---|---|---|---|---|---|---|---|---|---|---|---|---|---|---|---|---|---|
| | | | | | | | | | | | | | | | | | | | Cases/Controls with marker 1 and marker 2 conferring risk (1) or no-risk (0) | | | |
| Chr | Gene | Rs | P | OR (95% C.I.) | Minor/Major (risk) | PAF | Chr | Gene | Rs | P | OR (95% c.i.) | Minor/Major (risk) | PAF | OR (95% C.I.) | P | Permuted P | PAF | AUC | 00 | 01 | 10 | 11 |
| 4 | GRIA2 | rs17244157 | 0.07 | 1.17 (0.99:1.40) | G/A (G) | 0.128 | 16 | GRIN2A | rs1861192 | 0.41 | 1.05 (0.94:1.17) | A/G (A) | 0.087 | 0.44 (0.30:0.64) | 2.39E-5 | 0.85 | 0.120 | 0.5442 | 712/838 | 175/120 | 589/539 | 98/120 |
| 9 | SLC1A1 | rs10974611 | 0.80 | 1.02 (0.87:1.20) | T/C (T) | 0.121 | 6 | LAMA4 | rs2032568 | 0.33 | 1.05 (0.95:1.17) | G/A (G) | 0.072 | 0.47 (0.38:0.67) | 3.60E-5 | 0.92 | 0.125 | 0.5389 | 536/624 | 154/116 | 699/682 | 145/195 |
| 12 | GRIN2B | rs12371702 | 0.05 | 1.14 (1.002:1.31) | C/G ( C) | 0.113 | 9 | GRIN3A | rs13292935 | 0.77 | 1.03 (0.87:1.21) | T/C (T) | 0.126 | 0.41 (0.26:0.62) | 3.72E-5 | 0.95 | 0.114 | 0.5375 | 1003/1122 | 442/377 | 236/202 | 51/82 |
| 4 | GRIA2 | rs17244157 | 0.07 | 1.17 (0.99:1.40) | G/A (G) | 0.128 | 16 | GRIN2A | rs16966731 | 0.94 | 1.00 (0.91:1.11) | C/T ( C) | 0.065 | 0.45 (0.31:0.66) | 3.78E-5 | 0.95 | 0.127 | 0.5322 | 572/642 | 147/88 | 798/808 | 135/162 |
| 21 | GRIK1 | rs379182 | 0.02 | 1.96 (1.03:1.39) | G/A (G) | 0.131 | 11 | GRIK4 | rs17124632 | 0.39 | 0.95 (0.86:1.06) | A/G (G) | 0.084 | 0.24 (0.12:0.48) | 4.41E-5 | 0.97 | 0.148 | 0.5244 | 80/111 | 46/14 | 1165/1221 | 357/342 |
| 10 | RPP30 | rs11186343 | 0.19 | 1.14 (0.94:1.40) | C/T ( C) | 0.134 | 7 | HTR5A | rs2919435 | 0.50 | 1.03 (0.94:1.14) | A/G (A) | 0.057 | 2.49 (1.60:3.89) | 5.51E-5 | 0.98 | 0.130 | 0.5295 | 145/97 | 58/89 | 951/1028 | 558/555 |
| 3 | NR1I2 | rs6438549 | 0.74 | 1.03 (0.87:1.21) | A/G (A) | 0.126 | 12 | GRIN2B | rs12823982 | 0.55 | 1.04 (0.92:1.17) | C/T ( C) | 0.102 | 0.45 (0.30:0.67) | 7.17E-5 | 0.99 | 0.114 | 0.5333 | 927/1011 | 222/182 | 505/473 | 67/105 |
| 13 | HTR2A | rs666693 | 0.06 | 1.12 (1.00:1.26) | T/C (T) | 0.099 | 22 | COMT | rs8185002 | 0.33 | 0.95 (0.85:1.06) | G/T (T) | 0.087 | 3.05 (1.75:5.31) | 8.82E-5 | 0.99 | 0.148 | 0.5325 | 596/548 | 34/61 | 927/1011 | 83/58 |
| 4 | NR3C2 | rs7691663 | 0.64 | 0.98 (0.89:1.08) | C/A (A) | 0.052 | 5 | GRIA1 | rs1422897 | 0.93 | 1.00 (0.92:1.10) | A/C (A) | 0.049 | 2.09 (1.44:3.02) | 1.02E-4 | 0.99 | 0.110 | 0.5310 | 124/85 | 445/490 | 185/237 | 977/964 |
| 19 | GRIK5 | rs4803523 | 0.37 | 1.06 (0.94:1.19) | T/C (T) | 0.100 | 5 | GRIA1 | rs13359392 | 0.72 | 0.98 (0.89:1.08) | T/C ( C) | 0.066 | 2.31 (1.51:3.55) | 1.27E-4 | 1.0 | 0.144 | 0.5350 | 548/487 | 58/90 | 962/1068 | 161/135 |

D: Multiplicative recessive

| Marker 1 | | | | | | | Marker 2 | | | | | | | Interaction statistics | | | | | | | | |
|---|---|---|---|---|---|---|---|---|---|---|---|---|---|---|---|---|---|---|---|---|---|---|
| | | | | | | | | | | | | | | | | | | | Cases/Controls with marker 1 and marker 2 conferring risk (1) or no-risk (0) | | | |
| Chr | Gene | Rs | P | OR (95% C.I.) | Minor/Major (risk) | PAF | Chr | Gene | Rs | P | OR (95% c.i.) | Minor/Major (risk) | PAF | OR (95% C.I.) | P | Permuted P | PAF | AUC | 00 | 01 | 10 | 11 |
| 5 | GRIA1 | rs574071 | 0.03 | 0.88 (0.79:0.99) | A/G (G) | 0.144 | 11 | HTR3B | rs1672717 | 0.34 | 0.95 (0.86:1.05) | G/A (A) | 0.125 | 0.52 (0.39:0.71) | 2.72E-5 | 0.70 | 0.132 | 0.5433 | 371/473 | 701/646 | 222/182 | 305/346 |
| 4 | NR3C2 | rs7691663 | 0.64 | 0.98 (0.89:1.08) | C/A (A) | 0.124 | 5 | GRIA1 | rs9686702 | 0.90 | 0.99 (0.90:1.10) | A/G (G) | 0.131 | 1.84 (1.38:2.44) | 3.28E-5 | 0.76 | 0.117 | 0.5375 | 270/211 | 313/374 | 445/516 | 703/679 |
| 3 | GSK3B | rs1719889 | 0.02 | 0.87 (0.78:0.98) | A/T (T) | 0.145 | 16 | GRIN2A | rs1102967 | 0.32 | 0.95 (0.85:1.05) | T/C (C ) | 0.138 | 0.55 (0.41:0.73) | 3.30E-5 | 0.76 | 0.139 | 0.5423 | 228/345 | 569/517 | 308/289 | 534/552 |
| 11 | GRIA4 | rs17104807 | 0.03 | 0.81 (0.67:0.98) | G/A (A) | 0.149 | 22 | COMT | rs2020917 | 0.34 | 0.95 (0.85:1.06) | T/C (C ) | 0.140 | 2.39 (1.57:3.63) | 4.56E-5 | 0.86 | 0.118 | 0.5386 | 98/86 | 643/719 | 86/149 | 848/784 |
| 12 | GRIN2B | rs3764030 | 0.13 | 1.10 (0.97:1.24) | T/C (T) | 0.144 | 16 | GRIN2A | rs1868289 | 0.45 | 0.96 (0.87:1.06) | G/T (T) | 0.134 | 4.99 (2.26:11.03) | 6.95E-5 | 0.96 | 0.133 | 0.5169 | 41/13 | 29/44 | 695/742 | 948/970 |
| 5 | GRIA1 | rs574071 | 0.03 | 0.88 (0.79:0.99) | A/G (G) | 0.144 | 16 | GRIN2A | rs1070548 | 0.67 | 1.02 (0.92:1.13) | A/G (A) | 0.126 | 2.21 (1.48:3.31) | 1.17E-4 | 0.99 | 0.142 | 0.5284 | 517/532 | 819/860 | 70/110 | 186/135 |
| 9 | NTRK2 | rs1624327 | 0.02 | 0.88 (0.79:0.98) | A/G (G) | 0.142 | 16 | GRIN2A | rs1097784 | 0.66 | 1.02 (0.93:1.12) | T/C (T) | 0.116 | 1.94 (1.38:2.72) | 1.19E-4 | 0.99 | 0.138 | 0.5332 | 562/581 | 639/662 | 128/186 | 237/178 |
| 4 | ARHGAP10 | rs6824449 | 0.04 | 1.11 (1.01:1.22) | G/A (G) | 0.131 | 16 | SLC6A2 | rs192303 | 0.33 | 1.05 (0.95:1.63) | G/C (G) | 0.133 | 0.27 (0.14:0.54) | 1.68E-4 | 1.0 | 0.119 | 0.5319 | 1293/1414 | 208/159 | 184/144 | 16/32 |
| 6 | GRIK2 | rs7770500 | 0.68 | 0.95 (0.81:1.50) | G/A (A) | 0.150 | 6 | LAMA4 | rs2072019 | 0.98 | 1.00 (0.90:1.11) | A/G (G) | 0.070 | 2.06 (1.41:3.00) | 1.76E-4 | 1.0 | 0.116 | 0.5133 | 139/102 | 666/719 | 119/164 | 792/781 |
| 9 | SLC1A1 | rs7022369 | 0.14 | 1.07 (0.98:1.18) | C/G ( C) | 0.115 | 5 | GRIA1 | rs12658202 | 0.61 | 0.98 (0.89:1.07) | C/A (A) | 0.119 | 1.92 (1.36:2.71) | 1.98E-4 | 1.0 | 0.067 | 0.5285 | 100/147 | 281/326 | 399/352 | 957/952 |

**E: MDR**

| Marker 1 | | | | | | | Marker 2 | | | | | | |
|---|---|---|---|---|---|---|---|---|---|---|---|---|---|
| Chr | Gene | Rs | P | OR (95% C.I.) | Minor/Major (risk) | PAF (dom/rec) | Chr | Gene | Rs | P | OR (95% C.I.) | Minor/Major (risk) | PAF (dom/rec) |
| 12 | GRIN2B | rs1012587 | 0.14 | 1.09 (0.97:1.21) | A/T (A) | 0.095/0.142 | 3 | HTR3C | rs6762938 | 0.01 | 0.87 (0.79:0.96) | T/C ( C) | 0.061/0.128 |
| 16 | GRIN2A | rs8045893 | 0.01 | 1.19 (1.04:1.37) | C/T ( C) | 0.120/0.147 | 3 | HTR3C | rs6762938 | 0.01 | 0.87 (0.79:0.96) | T/C ( C) | 0.061/0.128 |
| 6 | HTR1E | rs17222848 | 0.27 | 1.08 (0.94:1.24) | T/A (T) | 0.115/0.148 | 5 | GRIA1 | rs17515709 | 0.97 | 1.00 (0.91:1.11) | G/C (G) | 0.063/0.133 |
| 11 | GRIA4 | rs609665 | 0.81 | 1.01 (0.92:1.12) | C/T ( C) | 0.058/0.131 | 6 | GRIK2 | rs2518171 | 0.06 | 1.11 (1.00:1.23) | A/G (A) | 0.079/0.136 |
| 6 | GRIK2 | rs17828670 | 0.05 | 1.16 (1.00:1.34) | G/T (G) | 0.123/0.148 | 3 | GSK3B | rs6771023 | 0.02 | 1.15 (1.02:1.30) | C/T ( C) | 0.106/0.145 |
| 13 | HTR2A | rs9526240 | 0.43 | 1.05 (0.94:1.17) | A/G (A) | 0.094/0.143 | 11 | BDNF | rs6265 | 0.14 | 0.92 (0.82:1.03) | T/C ( C) | 0.101/0.145 |
| 16 | SLC6A2 | rs1814270 | 0.75 | 1.02 (0.92:1.12) | C/T ( C) | 0.053/0.128 | 6 | FKBP5 | rs9462104 | 0.26 | 1.06 (0.95:1.19) | C/T ( C) | 0.083/0.138 |
| 13 | HTR2A | rs17288723 | 0.95 | 1.01 (0.87:1.16) | C/T ( C) | 0.113/0.147 | 9 | GRIN3A | rs11788456 | 0.60 | 0.98 (0.89:1.07) | G/A (A) | 0.043/0.121 |
| 12 | GRIN2B | rs10845847 | 0.52 | 0.97 (0.88:1.07) | A/C ( C) | 0.067/0.069 | 12 | GRIN2B | rs12301788 | 0.46 | 0.96 (0.87:1.07) | C/A (A) | 0.064/0.130 |
| 17 | SLC6A4 | rs2020939 | 0.41 | 1.04 (0.95:1.14) | A/G (A) | 0.031/0.108 | 6 | GRIK2 | rs2749074 | 0.02 | 1.12 (1.02:1.23) | T/C (T) | 0.048/0.119 |

| Interaction statistics | | | | | | | | | |
|---|---|---|---|---|---|---|---|---|---|
| | | | | Genotype combination at predicted affection status: | | Cases/Controls with genotype combination at predicted risk group | | | |
| Balanced accuracy | PAF | OR (95% C.I.) | P | High risk | Low risk | High risk | Low risk | TPR | FPR |
| 0.5513 | 0.0980 | 1.51 (1.32:1.73) | 1.168E-09 | AA/TT: AA/CT: AA/CC: AT/-2: TT/-2: TT/TT: TT/CT | -2/CT: -2/CC: AA/-2: AT/TT: AT/CT: AT/CC: TT/CC | 907/751 | 825/1032 | 0.524 | 0.421 |
| 0.5498 | 0.0900 | 1.49 (1.30:1.71) | 4.452E-09 | -2/TT: -2/CT: -2/CC: CT/-2: TT/-2: TT/TT: TT/CT | -2/-2: CC/-2: CC/TT: CC/CT: CT/TT: CT/CT: CT/CC: TT/CC | 1033/887 | 699/896 | 0.596 | 0.497 |
| 0.5480 | 0.0950 | 1.47 (1.28:1.68) | 1.707E-08 | -2/CG: -2/CC: TT/GG: TT/CC: AT/GG: AT/CC: AA/CG | -2/GG: TT/CG: AT/-2: AT/CG: AA/GG: AA/CC | 933/790 | 799/993 | 0.539 | 0.443 |
| 0.5476 | 0.0917 | 1.46 (1.28:1.68) | 2.051E-08 | -2/-2: -2/AA: -2/GG: CC/-2 CC/GG: CT/-2: CT/AA: CT/AG: TT/GG | -2/AG: CC/AA: CC/AG: CT/GG: TT/-2: TT/AA: TT/AG | 982/842 | 750/941 | 0.567 | 0.472 |
| 0.5470 | 0.0894 | 1.46 (1.28:1.67) | 2.757E-08 | -2/-2: -2/CC: -2/CT: GG/CT: GG/TT: GT/-2: GT/CC: TT/-2: TT/TT | -2/TT: GG/-2: GT/CT: GT/TT: TT/CC: TT/CT | 1021/884 | 711/899 | 0.589 | 0.554 |
| 0.5468 | 0.0912 | 1.45 (1.27:1.66) | 3.643E-08 | -2/CT: -2/CC: AA/TT: AA/CT: AG/TT: AG/CT: GG/-2: GG/CC | AA/CC: AG/CC: GG/TT: GG/CT | 984/847 | 748/936 | 0.568 | 0.475 |
| 0.5465 | 0.1020 | 1.46 (1.28:1.68) | 3.031E-08 | -2/CC: -2/TT: CC/-2: CT/CC: CT/CT: TT/CC: TT/TT | -2/CT: CC/CC: CC/CT: CC/TT: CT/-2: CT/TT: TT/-2: TT/CT | 809/668 | 923/1115 | 0.467 | 0.375 |
| 0.5454 | 0.0937 | 1.44 (1.26:1.64) | 8.039E-08 | -2/GG: -2/AA: CC/AG: CC/AA: CT/GG: CT/AA: TT/-2 TT/AG | -2/AG: CC/GG: CT/-2: CT/AG: TT/GG: TT/AA | 936/802 | 796/981 | 0.540 | 0.500 |
| 0.5454 | 0.0809 | 1.48 (1.29:1.71) | 2.458E-08 | -2/CC: -2/AC: -2/AA: AA/-2: AA/AC: AC/CC: AC/AC: AC/AA: CC/-2: CC/AA | AA/CC: AA/AA: AC/-2: CC/CC: CC/AC | 1180/1053 | 552/730 | 0.681 | 0.591 |
| 0.5448 | 0.1012 | 1.44 (1.26:1.65) | 8.982E-08 | -2/AG: -2/AA: CC/GG: CC/AG: CT/AG | -2/GG: CC/AA: CT/GG: CT/AA: TT/GG: TT/AG: TT/AA | 812/677 | 920/1106 | 0.469 | 0.380 |
