# Supplementary material for: The genetic interacting landscape of 63 candidate genes in Major Depressive Disorder: an explorative study
Source: BioData Min. 2014 Sep 9;7:19. doi: 10.1186/1756-0381-7-19 (PMC4181757; doi:10.1186/1756-0381-7-19)
Supplement: Additional file 5: Figure S2 — -Log10 P values plotted for the single markers tested in the STAR*D and GAIN samples. The 68 candidate genes are arranged in a relative scale along chromosome 1–22 and chromosome X. We performed a bioinformatic search and found no overlap between nominally associated markers in the STAR*D or GAIN samples with literature findings in the NHGRI GWAS catalog for risk to MDD. Nor were marker replicated in either the discovery or replication analysis in the mega-analysis of genome-wide association studies for major depressive disorder (Major Depressive Disorder Working Group of the Psychiatric GWAS Consortium Mol Psychiatry. 2013. doi:10.1038/mp.2012.21). However, two nominally associated markers in the GAIN sample were located in two genes (GRIA1 and GRIK3) which were found to be reported for risk to schizophrenia in NHGRI GWAS catalog. A: −Log10 of the P values are plotted for 654 genotyped markers in the STAR*D sample. The 10 nominally associated markers that were identical with markers genotyped in the GAIN dataset and included in the meta-analysis are highlighted in red. Threshold values for the nominal P value of 0.05 and Bonferroni’s corrected P value (7.6E-5) are illustrated. B: −Log10 of the P values are plotted for 1,516 genotyped (black) and 1,380 imputed (blue) markers in the GAIN sample. The 10 nominally associated markers that were identical with nominally associated markers in the STAR*D dataset and included in the meta-analysis are highlighted in red. Threshold values for the nominal P value of 0.05 and Bonferroni’s corrected P value (1.7E-5) are illustrated. C: −Log10 of the P values are plotted for the 10 markers tested in the meta-analysis. [file 1756-0381-7-19-S5.zip › 6384786221222972_add6_Table_S3B.doc]

| **Table S3 B:** Results of single marker analysis at P < 0.05 in GAIN | | | | | |
| --- | --- | --- | --- | --- | --- |
|  |  |  |  |  |  |
| **Chr** | **SNP** | **Gene** | **OR (95% C.I.)** | **P** | **Risk/Minor** |
| 6 | rs7751022 | HTR1E | 0.70 (0.87-0.57) | 0.0010 | A/A |
| 12 | rs11832404 | GRIN2B | 0.74 (0.90-0.62) | 0.0022 | C/C |
| 6 | rs6926133 | FKBP5 | 1.19 (1.35-1.06) | 0.0045 | C/A |
| 6 | rs2852583 | GRIK2 | 0.82 (0.94-0.71) | 0.0041 | G/G |
| 4 | rs4240338 | ARHGAP10 | 1.27 (1.51-1.07) | 0.0062 | C/T |
| 11 | rs2000870 | GRIK4 | 0.87 (0.96-0.78) | 0.0057 | G/G |
| 4 | rs9996108 | ARHGAP10 | 1.28 (1.53-1.07) | 0.0066 | A/G |
| 6 | rs2852584 | GRIK2 | 1.21 (1.39-1.05) | 0.0072 | T/A |
| 6 | rs6457836 | FKBP5 | 0.84 (0.96-0.74) | 0.0078 | T/T |
| 4 | rs11726983 | GRIA2 | 0.81 (0.95-0.69) | 0.0081 | A/A |
| 3 | rs6766410 | HTR3C | 0.88 (0.97-0.80) | 0.0093 | A/A |
| 1 | rs10917517 | HTR6 | 1.14 (1.26-1.03) | 0.0092 | C/C |
| 23 | rs12858300 | HTR2C | 1.35 (1.70-1.07) | 0.010 | G/C |
| 11 | rs11214790 | HTR3A | 1.20 (1.38-1.04) | 0.011 | G/T |
| 21 | rs446022 | GRIK1 | 1.21 (1.40-1.04) | 0.012 | A/G |
| 1 | rs10159394 | HTR6 | 1.13 (1.25-1.03) | 0.012 | G/A |
| 4 | rs17023870 | ARHGAP10 | 1.24 (1.48-1.04) | 0.014 | A/G |
| 11 | rs11604247 | HTR3A | 0.81 (0.96-0.69) | 0.014 | T/T |
| 1 | rs6658108 | HTR6 | 1.19 (1.37-1.04) | 0.014 | G/A |
| 22 | rs5749998 | MAPK1 | 1.12 (1.24-1.02) | 0.015 | T/T |
| 4 | rs10461267 | GRIA2 | 0.84 (0.97-0.73) | 0.016 | A/A |
| 6 | rs16878041 | HTR1E | 0.86 (0.97-0.75) | 0.016 | A/A |
| 4 | rs17244653 | GRIA2 | 1.21 (1.42-1.04) | 0.017 | C/T |
| 11 | rs7939968 | GRIK4 | 0.89 (0.98-0.81) | 0.017 | A/A |
| 4 | rs10018903 | ARHGAP10 | 0.81 (0.96-0.67) | 0.018 | A/A |
| 9 | rs3824473 | GRIN3A | 0.88 (0.98-0.79) | 0.018 | T/T |
| 22 | rs2283792 | MAPK1 | 0.89 (0.98-0.81) | 0.018 | T/T |
| 4 | rs4835456 | ARHGAP10 | 0.85 (0.98-0.75) | 0.021 | A/A |
| 22 | rs2298432 | MAPK1 | 1.12 (1.23-1.02) | 0.022 | C/A |
| 3 | rs1719889 | GSK3B | 1.15 (1.29-1.02) | 0.023 | T/A |
| 9 | rs10815017 | SLC1A1 | 0.89 (0.98-0.80) | 0.024 | T/T |
| 11 | rs4570590 | GRIK4 | 0.89 (0.99-0.81) | 0.026 | C/C |
| 9 | rs16920473 | RNF20 | 0.86 (0.98-0.75) | 0.026 | T/T |
| 4 | rs9332471 | ARHGAP10 | 0.81 (0.98-0.67) | 0.027 | C/C |
| 11 | rs17543669 | HTR3A | 1.24 (1.51-1.03) | 0.027 | T/C |
| 6 | rs828358 | HTR1E | 0.88 (0.99-0.78) | 0.029 | A/A |
| 9 | rs1885977 | RNF20 | 1.17 (1.34-1.02) | 0.029 | T/A |
| 5 | rs574071 | GRIA1 | 1.13 (1.27-1.01) | 0.031 | G/A |
| 11 | rs17104807 | GRIA4 | 0.81 (0.98-0.67) | 0.031 | G/G |
| 12 | rs219922 | GRIN2B | 1.11 (1.22-1.01) | 0.031 | C/T |
| 5 | rs503525 | GRIA1 | 0.86 (0.99-0.76) | 0.033 | T/T |
| 1 | rs12082412 | GRIK3 | 0.85 (0.99-0.74) | 0.033 | T/T |
| 5 | rs17706966 | HTR4 | 0.85 (0.99-0.73) | 0.035 | A/A |
| 4 | rs6824449 | NR3C2 | 1.11 (1.23-1.01) | 0.036 | A/G |
| 4 | rs11729081 | ARHGAP10 | 1.23 (1.50-1.01) | 0.038 | C/T |
| 6 | rs2023169 | GRIK2 | 0.90 (0.995-0.82) | 0.039 | C/C |
| 11 | rs12574754 | GRIA4 | 1.20 (1.44-1.01) | 0.040 | G/A |
| 11 | rs1938910 | GRIA4 | 1.20 (1.43-1.01) | 0.041 | A/A |
| 4 | rs11733409 | ARHGAP10 | 0.86 (0.99-0.74) | 0.043 | A/A |
| 6 | rs12215960 | GRIK2 | 0.86 (0.995-0.75) | 0.043 | G/G |
| 12 | rs7311110 | GRIN2B | 1.14 (1.29-1.004) | 0.043 | G/A |
| 4 | rs13124167 | ARHGAP10 | 0.86 (0.99-0.74) | 0.045 | C/C |
| 11 | rs4445646 | GRIK4 | 1.11 (1.22-1.003) | 0.045 | T/C |
| 3 | rs334535 | GSK3B | 0.89 (0.99-0.79) | 0.045 | T/T |
| 16 | rs12596924 | SLC6A2 | 0.88 (0.99-0.78) | 0.046 | G/G |
| 11 | rs7932158 | GRIK4 | 1.10 (1.21-1.0003) | 0.049 | T/T |
| 17 | rs1568447 | GRIN2C | 0.91 (0.999-0.82) | 0.049 | C/C |
